# Integral approach to organelle profiling in human iPSC-derived cardiomyocytes enhances *in vitro* cardiac safety classification of known cardiotoxic compounds

**DOI:** 10.3389/ftox.2025.1644119

**Published:** 2025-08-21

**Authors:** Brigitta R. Szabo, Jeroen Stein, Anna Savchenko, Thomas Hutschalik, Filip Van Nieuwerburgh, Tim Meese, Georgios Kosmidis, Paul G. A. Volders, Elena Matsa

**Affiliations:** ^1^ Ncardia Services B.V., Leiden, Netherlands; ^2^ Department of Cardiology, Cardiovascular Research Institute Maastricht (CARIM), Maastricht University Medical Center, Maastricht, Netherlands; ^3^ Department of Physiology, Cardiovascular Research Institute Maastricht (CARIM), Maastricht University Medical Center, Maastricht, Netherlands; ^4^ Laboratory of Pharmaceutical Biotechnology, Ghent University, Ghent, Belgium; ^5^ Cellistic, Mont-Saint-Guibert, Belgium; ^6^ Western Gateway Building, School of Biochemistry and Cell Biology, University College Cork, Cork, Ireland; ^7^ National Institute for Bioprocessing Research and Training (NIBRT), Dublin, Ireland

**Keywords:** hiPSC-derived cardiomyocytes, organelle profiling, cardiac safety, *in vitro* drug testing, fluorescence microscopy, cell painting, phenotypic screening

## Abstract

**Introduction:**

Efficient preclinical prediction of cardiovascular side effects poses a pivotal challenge for the pharmaceutical industry. Human induced pluripotent stem cell-derived cardiomyocytes (hiPSC-CMs) are becoming increasingly important in this field due to inaccessibility of human native cardiac tissue. Current preclinical hiPSC-CMs models focus on functional changes such as electrophysiological abnormalities, however other parameters, such as structural toxicity, remain less understood.

**Methods:**

This study utilized hiPSC-CMs from three independent donors, cultured in serum-free conditions, and treated with a library of 17 small molecules with stratified cardiac side effects. High-content imaging (HCI) targeting ten subcellular organelles, combined with multi-electrode array data, was employed to profile drug responses. Dimensionality reduction and clustering of the data were performed using principal component analysis (PCA) and sparse partial least squares discriminant analysis (sPLS-DA).

**Results:**

Both supervised and unsupervised clustering revealed patterns associated with known clinical side effects. In supervised clustering, morphological features outperformed electrophysiological data alone, and the combined data set achieved a 76% accuracy in recapitulating known clinical cardiotoxicity classifications. RNA-sequencing of all drugs *versus* vehicle conditions was used to support the mechanistic insights derived from morphological profiling, validating the former as a valuable cardiotoxicity tool.

**Conclusion:**

Results demonstrate that a combined approach of analyzing morphology and electrophysiology enhances *in-vitro* prediction and understanding of drug cardiotoxicity. Our integrative approach introduces a potential framework that is accessible, scalable and better aligned with clinical outcomes.

## 1 Introduction

Novel drug candidate development can take over a decade and exceed a billion dollars to reach the clinic (Hinkson et al., 2020), with cost of failure as one of the main culprits (Duyk, 2003). It is estimated that in recent decades, 90% of pharmaceutical development failure occurred at the clinical phase (Sun et al., 2022). Consequently, the urgency for pharmaceutical companies to address financially taxing, high drug attrition rates keeps increasing. Failure due to cardiac-safety complications is common (Waring et al., 2015; Sun et al., 2022), accounting for approximately one-third of adverse drug reactions leading to attrition (Fermini et al., 2018). According to the Food and Drug Administration’s (FDA) Adverse Event Reporting System for cardiovascular-related events, some of the most frequently listed categories include cardiac arrhythmia (often QT prolongation), cardiomyopathy, myocardial ischemia, and coronary-artery and valvular disorders (Laverty et al., 2011).

To this day, predictive models to determine the risk for these cardiovascular events with sufficient specificity and sensitivity are lacking. Numerous animal models have been utilized. However, their specificity at times can be low, resulting in ambiguous translational value (Lu et al., 2001; Bailey et al., 2014; Clark and Steger-Hartmann, 2018). Use of adult native cell types has also been plagued by low-efficiency and labor-intensive methods (Louch et al., 2011; Meki et al., 2021), in addition to their inadequate homogeneity, reproducibility, and *ex vivo* viability (Apati et al., 2019). Using an improved culture protocol of human heart slices, Miller et al. recapitulated clinically-observed cardiotoxic profiles of doxorubicin, trastuzumab, and sunitinib. However, the viability and functionality of these slices lasted only up to 6 days (Miller et al., 2020). In comparison, human induced pluripotent stem cell-derived cardiomyocytes (hiPSC-CMs) can be maintained in culture for months (Seibertz et al., 2023), making them suitable for investigating chronic compound effects and development of pathophysiology (Tiburcy et al., 2017).

The relevance of hiPSC-CM models as accessible, affordable, and scalable alternatives (Burridge et al., 2014; Fowler et al., 2020) in safety-pharmacology research has been demonstrated (Yamamoto et al., 2016; Ando et al., 2017; Blinova et al., 2017; Sharma et al., 2017; Blinova et al., 2018; Kopljar et al., 2018; Blinova et al., 2019; Giovannetti and Peters, 2021; Yang et al., 2022), especially in the context of detecting (pro)arrhythmic events (Yamamoto et al., 2016; Ando et al., 2017; Blinova et al., 2017; Blinova et al., 2018; Kopljar et al., 2018; Blinova et al., 2019). Major initiatives such as the Comprehensive *In Vitro* ProArrhythmia Assay (CiPA) have established protocols to study the short-term proarrhythmic effects of compounds using multi-electrode array (MEA) or voltage-sensitive dyes (VSD) by recording changes in electrophysiology, particularly focusing on the human ether-a-go-go-related gene (hERG) potassium channel modulation (Blinova et al., 2017; Blinova et al., 2018; Blinova et al., 2019). Despite high predictive power, such assays are unable to detect all types of cellular toxicities.

Most conventional screening assays focus only on a singular/few, readily interpretable functional parameters for analysis–such as alterations in action potential recordings–which can be directly correlated to specific biological functions or processes. While effective at precisely detecting certain abnormalities, these measurements may not fully capture the complexity of drug-induced cardiotoxicity, which requires a more integral approach. With the FDA phasing out animal testing for new drug candidates (Zushin et al., 2023) and the development of the EU Roadmap for Phasing Out Animal Experimentation (Walder et al., 2025), establishing integral *in vitro* cardiotoxicity models is timelier than ever. Through newly-emerging technologies, it is possible to analyze combinations of complex (sub)cellular morphological features, allowing for a more precise description of the sample condition (Chandrasekaran et al., 2021) – the morphological profiles generated are unique to the given condition in a similar fashion as a fingerprint would be to an individual. Current efforts such as the JUMP Cell Painting Consortium aim to standardize this approach and expand databases for enhanced drug safety assessments, however they are focused only on a few cell models including immortalized cancer cell lines, limiting their translational potential (Cimini et al., 2023; Chandrasekaran et al., 2024).

Incorporating novel morphological assays with established functional readouts could promote a cardiac safety paradigm shift towards combinatorial characterization, thereby improving the accuracy and resolution of hiPSC-CM models. In order to realize this, the present study relied on morphological profiling to accurately assess drug toxicity, facilitated by high-content imaging (HCI), a powerful tool for identifying potential drug targets and uncovering mechanisms of action (Bray et al., 2016; Pahl and Sievers, 2019). Quantitative data was extracted from acquired images of hiPSC-CMs stained by combinations of distinct markers for an array of ten subcellular organelles. This rich compilation of data was then utilized for clustering of potential cardio-toxicants, showing strong alignment with clinical classifications. The complementary nature of traditional functional assays, such as electrophysiology recordings to these readouts and *vice versa*, were investigated, demonstrating improved toxicity detection with the integrated approach. Moreover, RNA sequencing (RNA-seq) of compound-treated samples was performed to further elucidate the underlying transcriptional mechanisms of the cardiotoxic effects.

## 2 Materials and methods

### 2.1 hiPSC-CM plating and compound treatment

Cryopreserved hiPSC-CMs were thawed and precultured in fibronectin-coated (1:100) T75 flasks for 3 days, in Ncardia’s proprietary Cardiomyocyte Culture Medium, prior to being reseeded onto appropriate plate formats for compound assays. hiPSC-CM cultures were dissociated using 1X TrypLE™ Select Enzyme (Gibco™) and kept in serum-containing culture medium. On day 1, culture medium was switched to serum-free medium composition. hiPSC-CMs were maintained at 37°C and 5% CO_2_ with regular medium changes every 48 h. Only for the experiments relating to the assessment of the culture medium compositions, hiPSC-CMs were kept in culture for up to 14 days post seeding. After ascertaining the most suitable timepoint for all subsequent assays (day 8), all compound treatments began on day 7 post seeding, during which 3–6 replicates were exposed to one concentration of a single compound or to vehicle control (0.1% DMSO) for 24 h. Compound solutions were administered at final concentration of 0.1% DMSO (Table 1).

**TABLE 1:** Compound library and experimental concentration ranges used across assays.

Compound	Cmax (total)	Cexp	
		HCI and MEA	RNA-seq
Doxorubicin	1.3–6.8 µM (Twelves et al., 1991)	0.01–10 µM	0.1 µM
Cisplatin	6–18 µM (de Jong et al., 2023)	0.01–10 µM	10 µM
Ponatinib	0.05–0.18 µM (Cortes et al., 2012)	0.001–1 µM	1 µM
Dasatinib	0.15–0.37 µM (Takahashi et al., 2012)	0.001–1 µM	0.3 µM
Lapatinib	1.3–7.4 µM (Burris et al., 2005; Chu et al., 2007)	0.01–10 µM	10 µM
5-Fluoro-uracil	19.3–23 µM (Capitain et al., 2008)	0.01–10 µM	10 µM
Methotrexate	0.01–0.1 µM (Inoue and Yuasa, 2014)	0.001–1 µM	1 µM
Omecamtiv Mecarbil	22.4 nM–2.5 µM (Teerlink et al., 2011)	0.001–1 µM	1 µM
Propofol	21.9–56 µM (Fan et al., 1995; Bleeker et al., 2008)	0.1–100 µM	100 µM
Bupivacaine	1.6–5 µM (Hu et al., 2013)	0.01–10 µM	10 µM
Amiodarone	0.7–3.6 µM (Vassallo and Trohman, 2007)	0.01–10 µM	1 µM
Dofetilide	3.8 nM–23 nM (Allen et al., 2000)	0.0001–0.1 µM	0.01 µM
Digoxin	1–2.56 nM (Gona et al., 2023)	0.00001–0.01 µM	0.01 µM
Chlorpromazine	16 nM–560 nM (Rivera-Calimlim, 1982)	0.001–1 µM	1 µM
Erlotinib	1.2 nM - 5.9 µM (Gruber et al., 2018)	0.01–10 µM	0.3 µM
ASA	27–77 µM (Kanani et al., 2015)	0.01–10 µM	10 µM
Empagliflozin	∼665 nM (Scheen, 2014)	0.001–1 µM	1 µM

Summary of 17 reference compounds used in this study, including reported clinical maximum total plasma concentrations (Cmax) from literature, and experimental concentration ranges applied in high content imaging (HCI), multi-electrode array recordings (MEA) and RNA sequencing (RNA-seq). HCI and MEA assays were performed across a 7-point semi-logarithmic concentration range, while RNA-seq was conducted at a single selected concentration per compound.

### 2.2 Immunofluorescence staining and HCI protocol

Compound treated hiPSC-CMs seeded onto 384 well µClear black plates (Greiner) at a density of 2,000 cells per well, were stained for selected target structures. Live cell staining–MitoTracker CMXRos Red (25 nM) for 1 h; LysoTracker Red (75 nM) for 30 min at 37°C - was performed preceding fixation of samples using 4% methanol-free paraformaldehyde for 15 min at room temperature (RT). Next, cells were permeabilized with 0.01% Triton-X diluted in DPBS (−/−) for 15 min at RT. Primary antibody (Ab) mixes (Supplementary Table S1) were prepared in blocking solution containing 10% fetal bovine serum using 1:1,000 dilution scheme with the exception of the anti-PMP70 which was diluted to 1:500. Samples were incubated overnight at 4°C then washed thrice with 0.01% Triton-X. Appropriate secondary Ab mixes (1:500 dilution) were added for 2 h at room temperature. Concanavalin (200 μg/mL) staining for 1 h and Wheat Germ Agglutinin (5 μg/mL) for 10 min preceded nuclear staining with DAPI (1:1,000, 15 min at RT). Lastly, cells were washed thrice with 0.01% Triton-X as well as with DPBS (−/−).

High-magnification images (40×) were acquired using an ImageXpress Micro Confocal platform (Molecular Devices) in confocal mode. Images were analyzed in MetaXpress software version 6.6, using the Custom Module Editor, in which unique masks were designed to detect each subcellular component on a single-cell level (Supplementary Figure S1). Signals corresponding to nucleoli (anti-fibrillarin Ab) and DNA damage (anti-γH2AX Ab) were detected within the area corresponding to nuclei (DAPI), and remaining organelles within the segmented cytoplasmic area. During image analysis, the fluorescence threshold was not set as an absolute value for signal quantification but as an intensity difference to local background ensuring comparable noise to signal ratio for each dataset of an immunofluorescence staining experiment.

### 2.3 RNA-sequencing experimental procedure

On day 8, post 24-h compound treatment cell samples were collected from hiPSC-CMs for RNA extraction. For each compound, the treatment concentration was selected as the dose that elicited the largest functional effect on MEA recordings without causing significant loss of viability (Table 1), so as to not bias mechanistic analysis towards cell death pathways and allow elucidation of causative toxicity mechanisms. RNA was isolated using the NucleoSpin RNA kit (Bioke) according to the manufacturer’s protocol.

After RNA extraction, the concentration and quality of the total extracted RNA were evaluated by using the “Quant-it ribogreen RNA assay” (Life Technologies) and the RNA 6000 Nano chip (Agilent Technologies), respectively. Subsequently, 10 ng of RNA was used to perform an Illumina sequencing library preparation using the QuantSeq 3′ mRNA-Seq Library Prep FWD Kit (Lexogen) per manufacturer’s instructions. During library preparation, 17 PCR cycles were used. Libraries were quantified by qPCR, according to Illumina’s protocol ‘Sequencing Library qPCR Quantification protocol guide’, version February 2011. A High Sensitivity DNA chip (Agilent Technologies) was used to control the library’s size distribution and quality. Sequencing was performed on a high throughput Illumina NextSeq 500 flow cell generating 75 bp single reads.

### 2.4 Bioinformatic analysis

#### 2.4.1 Imaging dataset

**FIGURE 1:**
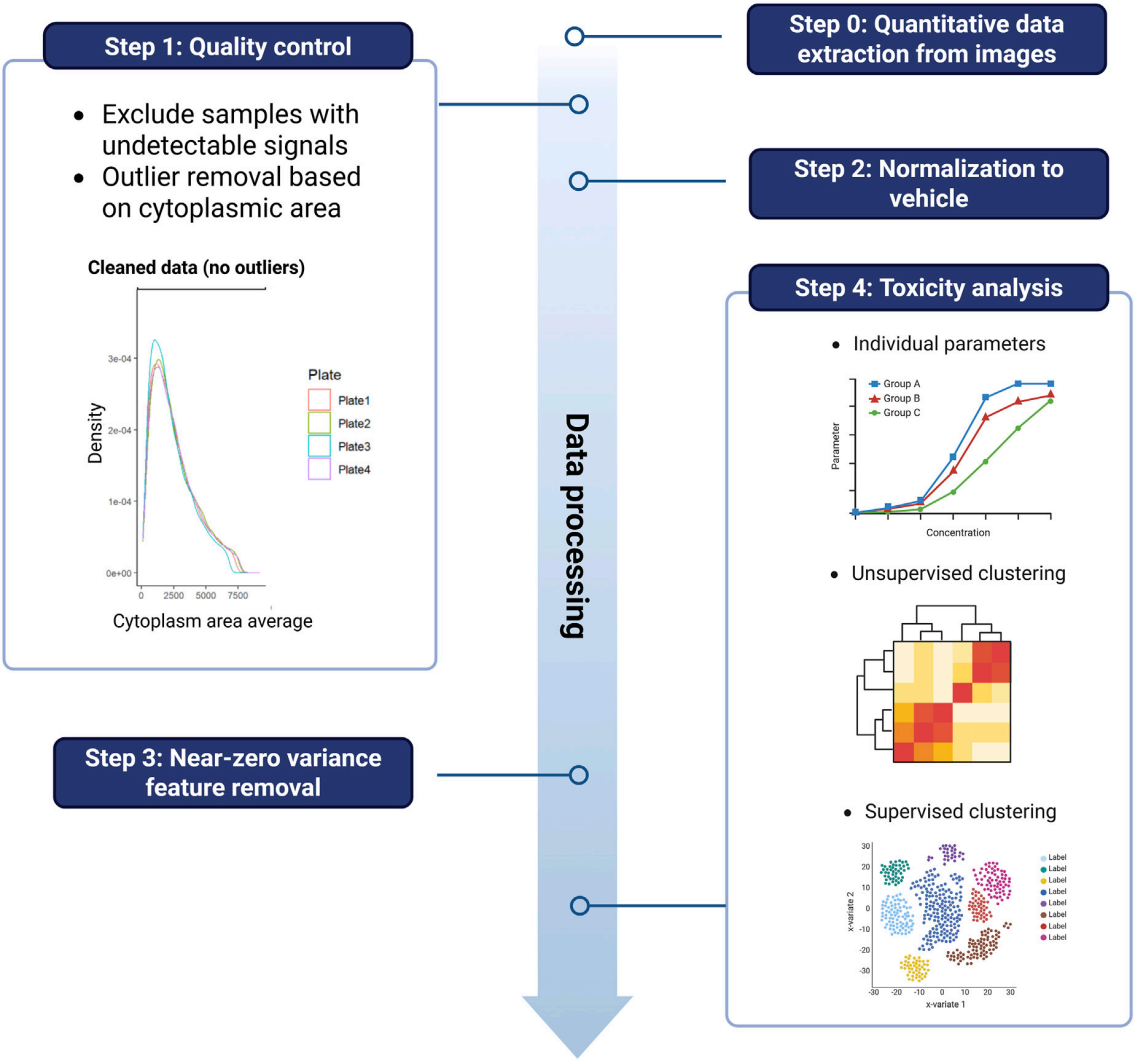
Stepwise workflow of imaging-derived feature processing used for unsupervised and supervised toxicity classification.

Imaging-derived features exported from MetaXpress software as. txt files were analyzed using R studio (R v.4.2.3) following the steps detailed below (Figure 1). Quality control involved exclusion of conditions with undetectable signals, as well as outlier filtering based on cytoplasmic area as correct detection of cytoplasmic area served as the basis of further cell segmentation steps. Outlier filtering was performed using a median absolute deviation (MAD) thresholding approach. For each dataset (i.e., cell line/batch and staining protocol), cells were flagged as outliers if their cytoplasmic area deviated from the median by more than three MADs. A threshold of three MADs was applied in alignment with statistical practices and to ensure conservative exclusion of extreme values in biologically variable datasets (Leys et al., 2013). Specifically, for each value (*x*) the outlier condition was defined as:

$$\left|\frac{x-median\;\left(X\right)}{MAD\left(X\right)}\right|>3$$

where X is the vector of all values in that dataset. In cases where all values were identical (i.e., MAD = 0), no outliers were removed, as cells were assumed to exhibit similar morphology. Additionally, to avoid errors by dividing zero by zero, any mathematically undefined results were treated as non-outliers as well. This approach ensured robust and reproducible outlier handling across all experimental batches.

Post-filtering, data were aggregated at the replicate-level by calculating median values across all cells per replicate. Each replicate-level profile was then normalized to the median of the corresponding vehicle control. Non-informative features with near zero variance were identified and excluded from further analysis. Near zero variance was classified as meeting both of the following criteria: a high frequency ratio (default threshold: >19, i.e., the most common value occurred 19 times more often than the second most common value), and a low proportion of unique values (<10%). These thresholds were applied across the whole dataset to identify predictors with limited discriminatory value.

For unsupervised analysis (principal, component analysis; PCA), cell line-level aggregated data was used to identify broad phenotypic trends across donors. In addition, to capture phenotypic signals emerging at any level of exposure and to derive global conclusions, all seven concentrations tested per compound were pooled and treated as a single input group. This strategy allowed for the detection of emergent morphological patterns across all treatment levels without bias from donor-specific variability in dose-response thresholds. Absolute loading scores from principal component 1 (PC1) were averaged across lines to identify key features and generate compound-level toxicity clusters *via* k-means clustering.

In contrast, supervised classification was conducted using sparse partial least squares discriminant analysis (sPLS-DA) on replicate-level data from a single, well-characterized hiPSC-CM batch (Ncyte CM1). Similarly, as for RNA-seq, one concentration per compound was selected based on the strongest functional (MEA) effect without loss of viability (Table 1). This design minimized technical variability and enabled high-resolution compound discrimination and classification aligned with known clinical toxicity groupings.

All statistical analyses and visualizations were performed using established R packages: tidyverse (v.2.0.0), dplyr (v.1.1.4), caret (v.6.0.94), mixOmics (v.6.25.1), ggplot2 (v.3.5.1) and pheatmap (v.1.0.12).

#### 2.4.2 Differential gene expression

Per sample, on average 5.7 × 10^6^ ± 1.3 × 10^6^ reads were generated. First, raw reads were quality and adapter trimmed with Trim Galore v.0.6.7. The trimmed reads were mapped against the human genome (GRCh38.104) with STAR v.2.7.9a. (Dobin et al., 2013). Unique Molecular Identifiers were used during the sequencing and were processed with UMI-tools v.1.1.2. (Smith et al., 2017). The RSEM software v.1.3.1 (Li and Dewey, 2011) was used to generate the count tables.

Differential gene expression–in which the vehicle group was compared to a single compound group–was performed using edgeR v.3.36.0. (Lun et al., 2016). For each separate analysis, included the following: (1) Normalization using edgeR’s standard normalization method. (2) Removing low expressed genes with the filterByExpr function. (3) A general linear model was built with an empirical Bayes quasi-likelihood F-test to identify genes as significantly different if FDR ≤0.05 and FC ≥ 1.

Genes were annotated for ontologies using DAVID (Huang da et al., 2009) in conjunction with the GOplot v.1.0.2 R package.

### 2.5 Electrophysiology analysis

hiPSC-CMs were seeded in a droplet (10,000 cells/droplet), covering all electrodes of fibronectin (1:20) coated Axion CytoView 96 well plates. Cultures were maintained at 37°C, 5% CO_2_, with all plates equilibrated for 30 min prior to recordings. Electrophysiological parameters were obtained from field potential recordings of contracting hiPSC-CMs using an Axion Maestro Pro device and the corresponding Axis Navigator and Cardiac Analysis tool. Plates were recorded for 5 min. Parameters analyzed included; active electrodes (number of electrodes detecting beats in percentage with beat detection set at 300 µV), beat rate (beats per minute; BPM), beat rate irregularity (coefficient of variation within each well between the eight electrodes, BRI), beat rate variability (coefficient of variation in between wells of the same plate; BRV CV), field potential duration (ms; FPD), corrected field potential duration (FPDc), FPD detection success rate (percentage of active electrodes which could also detect FPD successfully), FPD variability (coefficient of variation in between wells of the same plate; FPD CV). Calculations were performed following the equations listed:

$$BPM=\frac{60\;\left(s\right)}{Beat\;period\;\left(s\right)}$$

$$BRI=\frac{Standard\;deviation\;\left(SD\right)\;beat\;period\;per\;electrode}{Mean\;beat\;period\;per\;well}x\;100$$

$$BRV\;CV=\frac{Standard\;deviation\;\left(SD\right)\;BPM\;per\;well}{Mean\;BPM\;per\;plate}x\;100$$

FPDc=FPD msBeat period s^0.192

$$FPD\;CV=\frac{Standard\;deviation\;\left(SD\right)\;FPD\;per\;well}{Mean\;FPD\;per\;plate}x\;100$$

### 2.6 Data processing and visualization

Prism GraphPad software was used for additional data processing. Error bars on graphs represent the SEM indicating the precision of the estimated population mean, or SD indicating the data variability around the mean.

## 3 Results

### 3.1 Serum-free medium enhances hiPSC-CM metabolic function and electrophysiological stability

To ensure predictable compound action, high reproducibility, and more physiologically relevant function for hiPSC-CMs, such as a more mature metabolic phenotype (Feyen et al., 2020), several chemically defined, serum-free hiPSC-CM culture medium compositions were tested as alternatives to standard serum-containing medium (Supplementary Table S2).

The effects of media formulations on cellular electrophysiology were one of the parameters used to assess their suitability. Synchronized electrical activity of cardiomyocytes is essential for producing effective contractions and physiological function in the heart. Thus, propagation patterns of electrical impulses in spontaneously contracting monolayers of Ncardia’s proprietary bioreactor-derived ventricular-like hiPSC-CMs (Ncyte^®^ vCardiomyocytes, Ncyte CM1) were monitored from the first day of consistent spontaneous activity, i.e., day 4 until day 14, in all media using MEA (Supplementary Figure S2A).

**FIGURE 2:**
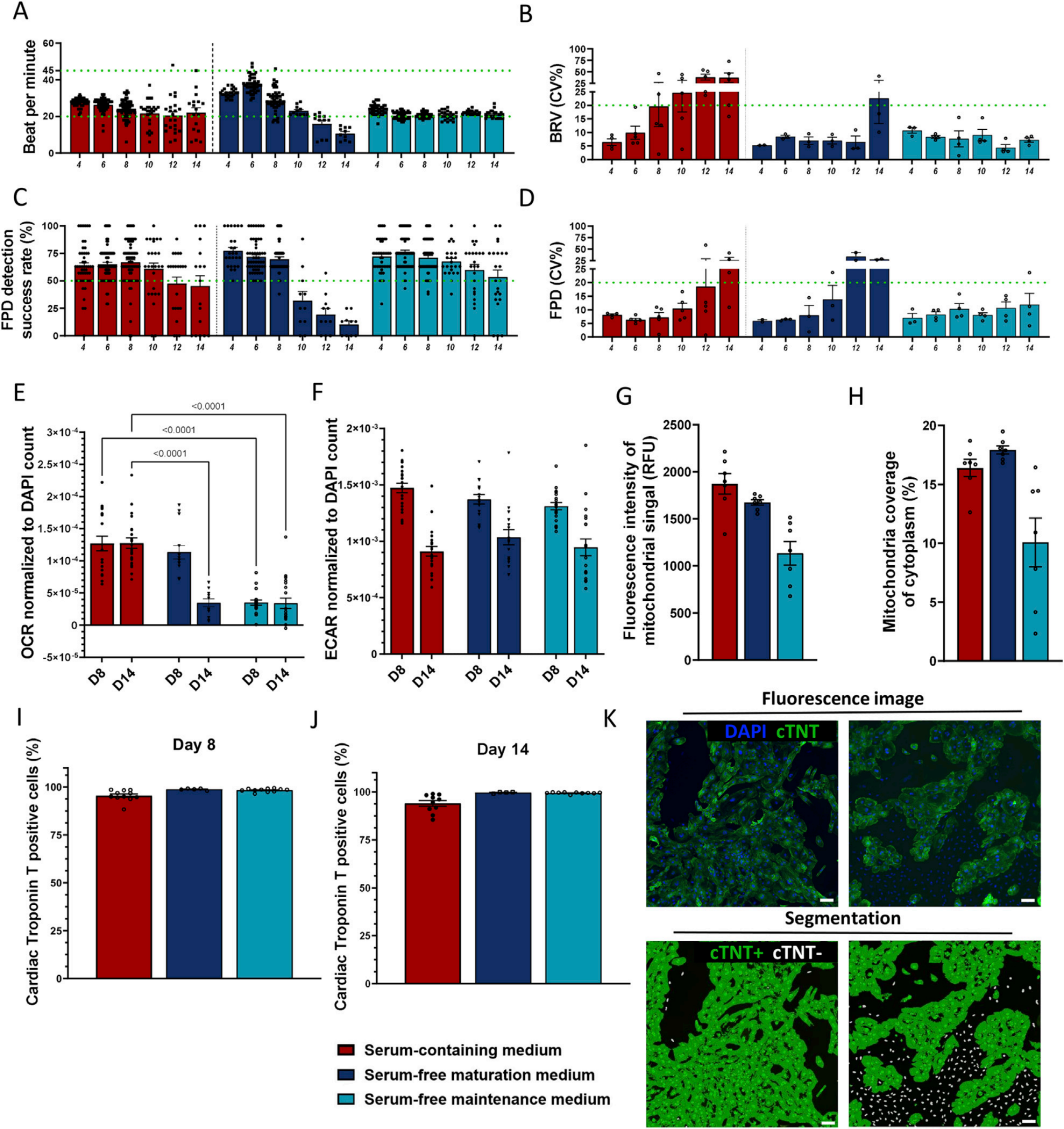
Evaluation of two serum-free media formulations supplemented with 0.5% BSA +1% KOSR. **(A–D)** Electrophysiological parameters of hiPSC-CMs measured in media that passed the initial screening; **(A)** beats per minute (BMP; threshold 20–45), **(B)** beat rate variation (BRV, inter-well variability coefficient of variation, threshold ≤20%), **(C)** field potential duration (FPD) detection success rate (threshold ≥50%) and **(D)** FPD variation (inter-well variability coefficient of variation, threshold ≤20%). **(E,F)** Metabolic activity assessed at days 8 and 14 post-seeding *via* **(E)** oxygen consumption rate (OCR) and **(F)** extracellular acidification rate (ECAR), both normalized to DAPI stained nuclei (µs/h/cell). One-Way ANOVA between control *versus* serum-free media per time point, p < 0.05 shown. **(G,H)** Assessment of mitochondrial network using MitoTracker staining on day 8. **(G)** Fluorescence signal intensity (absolute values), **(H)** area of the total cell cytoplasm covered by mitochondrial signal. **(I–K)** Cardiac troponin T positive cells (%) **(I)** on day 8 **(J)** on day 14. **(K)** Representative fluorescence images of high-purity (right panel) and low-purity (left panel) cultures stained for nuclei (blue) and cTNT (green). Segmentation mask showing cTNT-positive cytoplasm (green) and cTNT-negative nuclei (white). Scale bar = 100 µm. Data represented as mean ± SEM, N ≥ 3 (biological repeats from Ncyte CM1), with n ≥ 10 (technical replicates) for all timepoints, except for mitochondrial immunofluorescence measurements N = 1, n = 8.

Conditions in which higher rate of decreased electrical activity of hiPSC-CMs (i.e., active electrodes <50%) and arrhythmic contractions–asynchronous contractions between the eight electrodes within each well (i.e., beat-rate irregularity (BRI) >5%) were excluded (Supplementary Figure S2B). The combination of bovine serum albumin (BSA) and Knock-out Serum Replacement (KOSR) enabled stability (Figure 2A–D; Supplementary Figure S2B; time period from day 4 to 14) and metabolic function (Figures 2E–H) comparable to the serum-containing control medium. As expected, several electrophysiological parameters changed over time across the tested media conditions. In the serum-containing control medium, the percentage of active electrodes began to decline after day 8 (Supplementary Figure S2B), accompanied by increased variability in beat rate (BRV CV) in-between individual wells cultured in the same medium and a downward trend in beats per minute (BPM) (Figures 2A,B). The BSA + KOSR-supplemented maturation medium showed similar patterns, nevertheless the timing of decline was delayed. Furthermore, the BSA + KOSR-supplemented maintenance medium maintained stable values for active electrodes, BPM, and BRV CV up to day 12, indicating the most prolonged electrophysiological stability and enabling a stable assay window until day 14 (Figures 2A–D). This data supported the selection of day 8 as the most stable timepoint for further assays (Supplementary Figure S2A).

Cardiomyocytes sustain their electrical activity and physiological functions through an abundant network of mitochondria, which require a continuous supply of respiratory substrates to meet their high energy demands (Li et al., 2020; Yang et al., 2023). Disruption in the ATP-generating pathways or alterations in the mitochondrial network significantly impacts cardiac functionality (Brown et al., 2017). These characteristics can also serve as critical markers for the maturity of hiPSC-CMs. To select which candidate culture condition could facilitate an adult-like metabolic state, metabolic shift from glycolysis to fatty acid oxidation–a hallmark of adult cardiomyocytes (Ahmed et al., 2020) – was monitored. Extracellular acidification rate (ECAR), i.e., cellular glycolysis, decreased over time in all media (Figure 2F), suggesting that prolonged cell culture promotes maturation towards an adult like state. Supplemented serum-free maturation medium showed comparable oxygen consumption rate, as measured by oxidative phosphorylation rate (OCR), to serum-containing medium on day 8 (Figure 2E). However, by day 14, the OCR decreased significantly (p < 0.001). hiPSC-CMs cultured in supplemented serum-free maintenance medium displayed the lowest OCR at all time points (3.5 × 10^−5^ ± 3.9^–6^ and 3.4 × 10^−5^ ± 8.1^–6^). As the OCR remained at similar levels with the serum containing media up to day 8, this was chosen as endpoint. Quantitative morphological assessment of mitochondria was consistent with OCR results, showing higher mitochondrial signal intensity (1,674 ± 28.87 vs. 1,134 ± 125.6 RFU) and area coverage (17.93% ± 0.34% vs. 10.07% ± 2.06) in maturation medium (Figures 2G,H)*.*

Cardiac-specific marker troponin T (cTNT) immunostaining at all time points reached cTNT positivity ≥95% (Figures 2I–K), indicating that serum-free media did not prompt preferential growth of non-cardiomyocyte cells.

Maturation promoting medium supplemented with 0.5% total BSA plus 1% total KOSR was selected for further experiments, due to its beneficial effects on metabolism.

### 3.2 Morphological profiling detects cardiotoxicity and provides mechanistic insight to side effects

Subsequently, to enable scalable morphological profiling of compound-induced cardiotoxicity, high-content imaging (HCI) protocols were optimized for detecting structural changes in subcellular organelles. In comparison with the traditional cell painting assay (Bray et al., 2016; Pahl and Sievers, 2019; Cimini et al., 2023) the panel of fluorescent dyes and antibodies was expanded (Supplementary Table S1). Staining protocols targeting DNA damage (γH2AX), sarcomere (cTNT), gap junctions (connexin 43, CX43), nucleoli, mitochondria, lysosomes, peroxisomes, the Golgi apparatus and endoplasmic reticulum (ER) were validated in a multiplexed 384-well format, forming the basis of our custom organelle-profiling assay.

**FIGURE 3:**
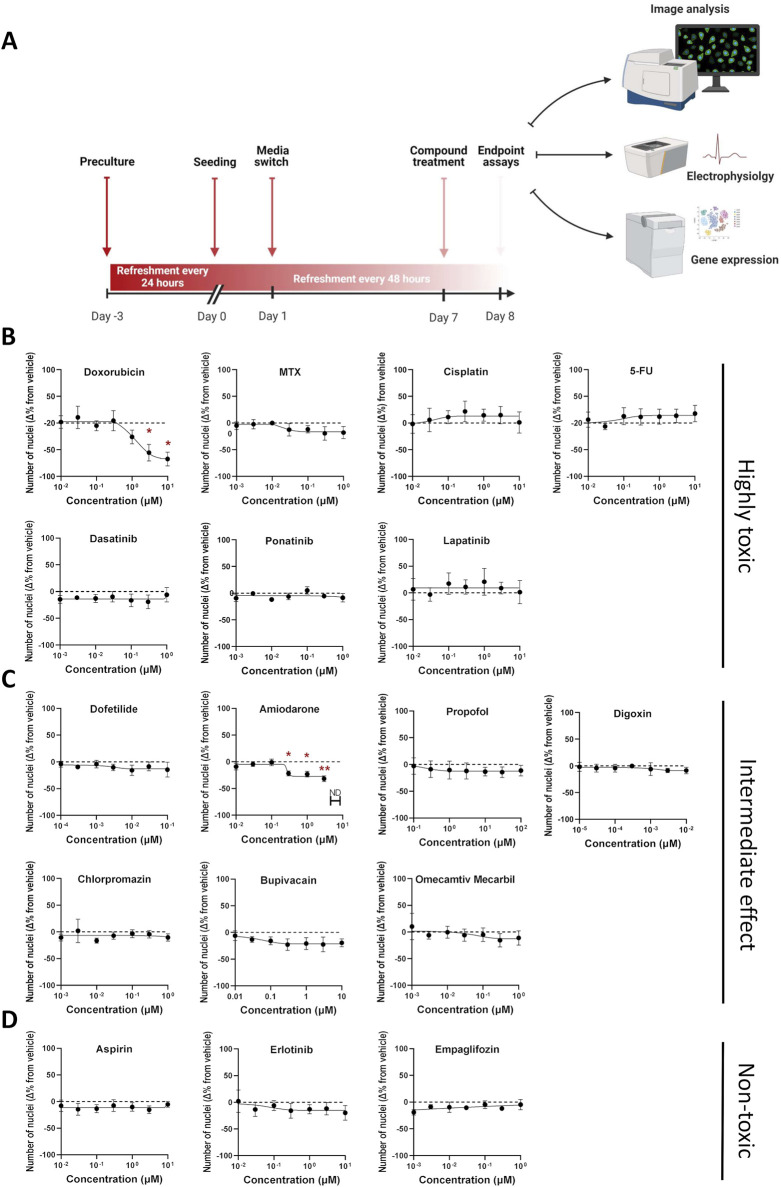
Assessment of compound-induced effects on cell viability in hiPSC-CMs. **(A)** Schematic outline of the experimental design. **(B–D)** Percentage change in cell viability following 24-h compound treatment, relative to DMSO vehicle control. Cell viability was estimated based on the number of DAPI-stained nuclei per well (nine fields per well). Data are presented as mean ± SEM; N = 4 (biological replicates from different hiPSC-CM lines), with n = 6 (technical replicates per condition). Statistical significance was assessed using Kruskal–Wallis test comparing each compound to vehicle; only significant p-values are displayed (*p < 0.05, ** p < 0.01, *** p < 0.001, **** p < 0.0001).

Protocol optimization involved comparing single *versus* multiplexed staining strategies using well-characterized positive control compounds for each organelle (Supplementary Figure S3). For instance, since doxorubicin is associated with DNA damage, as well as gap junction and nucleoli defects, corresponding staining protocols were tested. Initial nucleolar visualization employed SYTO14, a nucleic acid dye with a broad excitation/emission spectrum. However, due to significant channel bleed-through in multiplexed conditions, doxorubicin-induced nucleolar alterations were only detectable in single-stain formats. Consequently, SYTO14 was excluded from the final protocol and replaced by a fibrillarin antibody as a more specific nucleolar marker to ensure reliable detection in multiplexed imaging. Further organelle-specific responses were confirmed by known mitochondrial toxicants e.g., rotenone (Won et al., 2015) and chloroquine (Javaid et al., 2022). Rotenone reduced mitochondrial area and number, similarly to chloroquine, which also increased fluorescence intensity of the mitochondrial signal. Further chloroquine is also a lysosomotropic agent that increases pH by accumulating within these organelles as a deprotonated weak base and blocking the binding of autophagosomes to lysosomes (Homewood et al., 1972; Jia et al., 2018), which in this study led to increased lysosomal area and decreased lysosomal fluorescence intensity. Thapsigargin and brefeldin A, elevated ER and Golgi marker signals respectively, while H_2_O_2_, at all tested concentrations (10, 100 µM), induced a mild reduction in peroxisomal signal. Overall, except for SYTO14, compound-induced changes were comparable across single and multiplexed protocols, validating the robustness of the latter for scalable, phenotypic profiling in hiPSC-CMs. This multiplexed protocol was used in all subsequent compound screening assays studying the effects of a curated library of compounds (Figure 3A).

Seventeen reference compounds were selected from three categories: i) seven highly toxic chemotherapeutic agents from different drug classes ii) seven intermediate/unknown-; antiarrhythmics, anesthetics, antipsychotics, and a cardiac myosin activator iii) three non-toxic compounds; one tyrosine kinase inhibitor (TKI), one platelet aggregation inhibitor and one anti-diabetic agent. This selection was made to capture a broad spectrum of mechanisms, from well-known cardiotoxic effects to less understood or non-toxic profiles. Each compound was tested across a 7-point semi-logarithmic concentration range designed to span the reported clinical maximum total plasma concentration (Cmax) values, when feasible, and to balance clinical relevance with detection of perturbations across a range of exposure levels (Table 1). To counter line-to-line variability, studies were performed in three different hiPSC-CM lines generated using 2D monolayer (NC196) or 3D bioreactor (2 batches of Ncyte CM, NCRM5) differentiation protocols (Supplementary Table S3). All hiPSC-CMs showed high expression of standard cardiomyocyte markers prior to being treated with the compounds listed above (Supplementary Figure S4).

Experiments confirmed that compounds listed as non-toxic, showed no changes in viability between treated samples and concentration-matched DMSO controls. In contrast, across all hiPSC-CMs, significant reduction in nuclear count was observed for doxorubicin and amiodarone. Specifically, doxorubicin reduced the nuclei count by 67% ± 13 at 10 µM (Figure 3B), whereas the same concentration of amiodarone led to a maximal reduction of 31% ± 5 (Figure 3C). Nuclei count served to assess cardiomyocyte numbers. A secondary assay to verify changes to viability was conducted *via* measuring DNA content (Supplementary Figure S5). It should be noted that actual number of hiPSC-CMs and DNA content could slightly differ by the ratio of multi-nucleated cells which is typically in the range of 8%–20% (da Rocha et al., 2017; Woo et al., 2019).

**FIGURE 4:**
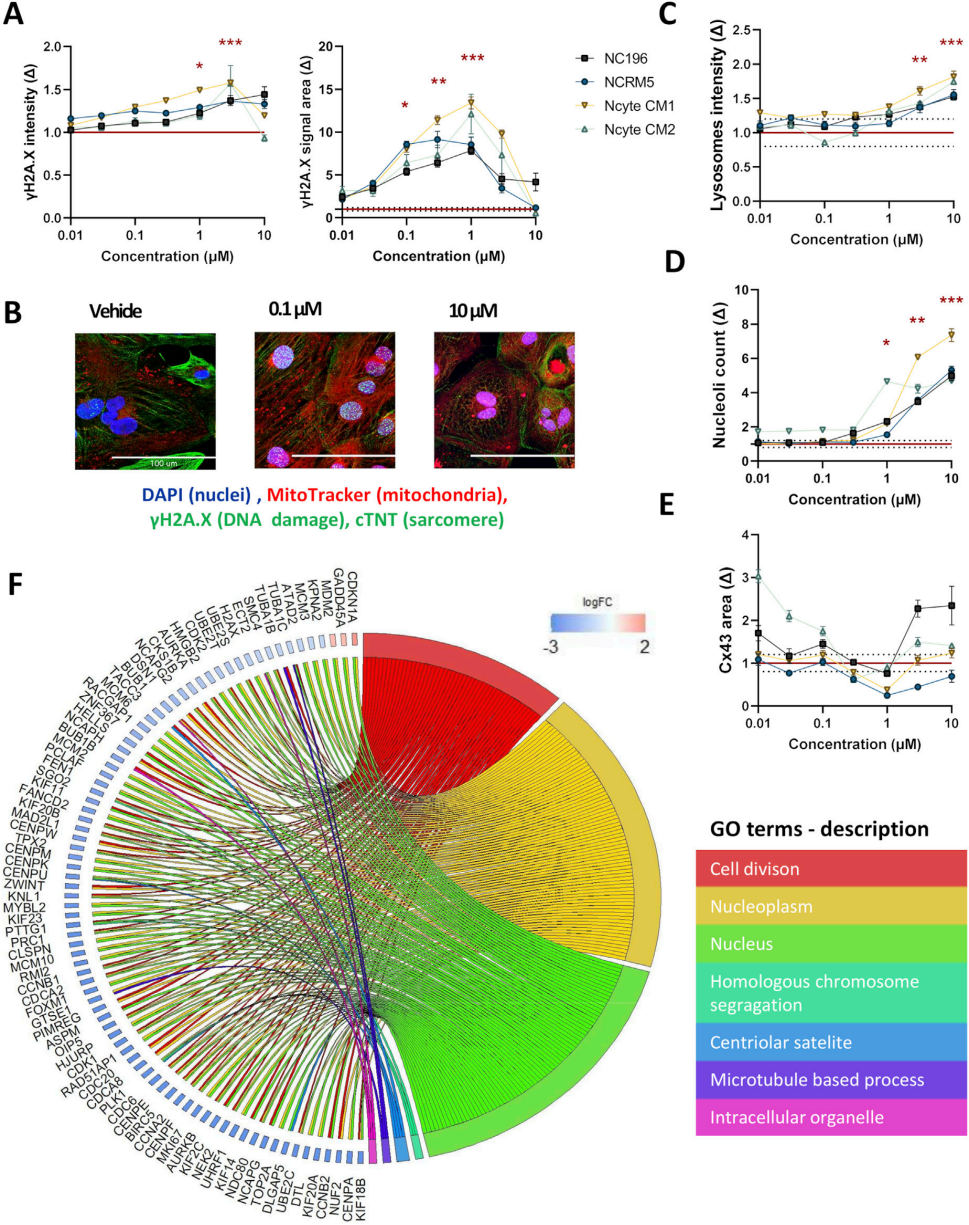
High-content imaging and transcriptomic profiling of doxorubicin-treated hiPSC-CMs. **(A)** γH2AX staining was used to detect DNA damage following doxorubicin treatment. Fluorescence intensity and area are shown per hiPSC-CM line across a 7-point semi-logarithmic concentration range. **(B)** Representative 40X images of γH2AX (within the nuclei), troponin (cytoplasm), mitochondria and nuclei staining. Scale bar = 100 µm. **(C–E)** Representative morphological changes in **(C)** lysosomal intensity, **(D)** nucleoli count, and **(E)** gap junction area following compound treatment. **(F)** Gene Ontology (GO) enrichment analysis of RNA-seq data from hiPSC-CMs treated with 0.1 µM doxorubicin. Displayed are non-redundant GO terms (biological process, cellular component, molecular function) with at least three differentially expressed genes and genes involved in two or more categories, along with corresponding logFC2 (effect size) values for included genes are shown. Data are presented as mean fold change (Δ) relative to vehicle ±SEM. High-content imaging: N = 4 (biological replicates from different hiPSC-CM lines), n ≥ 6 (technical replicates per condition). RNA-seq: N = 3 (Ncyte CM1). Statistical significance assessed by Kruskal–Wallis test; p < 0.05 displayed (*p < 0.05,** p < 0.01, *** p < 0.001, **** p < 0.0001).

In the morphological profiling analysis, doxorubicin induced the most pronounced and widespread changes across organelle-specific readouts. γH2AX, a well-established marker of early cellular response to DNA damage and genotoxic stress (Li et al., 2008) showed a clear dose-dependent increase in both fluorescence intensity and area–detected within the nuclear area of the cells–across hiPSC-CM lines. Statistically significant increases in γH2AX intensity were observed at 1 μM and 3 µM (Ncyte CM1: 1.6 ± 0.05, Ncyte CM2: 1.5 ± 0.5, NCRM5: 1.4 ± 0.04, NC196: 1.3 ± 0.14) while nuclear area was significantly elevated from 0.1 µM to 1 µM (Ncyte CM1: 13.4 ± 1.7, Ncyte CM2: 12.1.6 ± 5.2, NCRM5: 8.5 ± 2.1, NC196: 7.9 ± 1.1) (Figures 4A,B). Interestingly, at the highest concentrations, γH2AX signal intensity decreased, likely reflecting progressive cell death rather than diminished DNA damage. This biphasic response is consistent with γH2AX’s role as an early stress marker, preceding overt cytotoxicity.

To further investigate the transcriptional effects underlying the observed DNA damage response, RNA sequencing (RNA-seq) was performed using cells treated with doxorubicin at 0.1 µM (Table 1). At this concentration, selected as having shown phenotypic changes but no cell death that would cloud gene ontology (GO) analysis, a total of 125 genes were differentially expressed compared to vehicle controls, with the majority being downregulated. GO term analysis revealed significant enrichment for pathways related to cell division and nuclear organization, including downregulation of genes involved in mitotic regulation, such as cyclin-dependent kinases (e.g., CDK1) and associated regulators. Notably, FAM111B, a protease implicated in DNA repair and cell survival (Arowolo et al., 2022) showed the largest effect size among all differentially expressed genes. Importantly, TOP2A, the molecular target of doxorubicin and key component of DNA replication and repair was significantly downregulated (logFC: −5.29) (Figure 4F).

Further known toxicities of doxorubicin were detected by imaging, namely; significant increase in lysosomal intensity (p < 0.05 at 3 μM, 10 µM) and nucleolar number (p < 0.05 at 1 μM–10 µM), changes in CX43 area could also be observed, however this effect showed high variability (Figures 4C–E). Differences observed in sensitivity across cell lines highlight the importance of including multiple cell lines with individual genetic backgrounds in compound screening.

**FIGURE 5:**
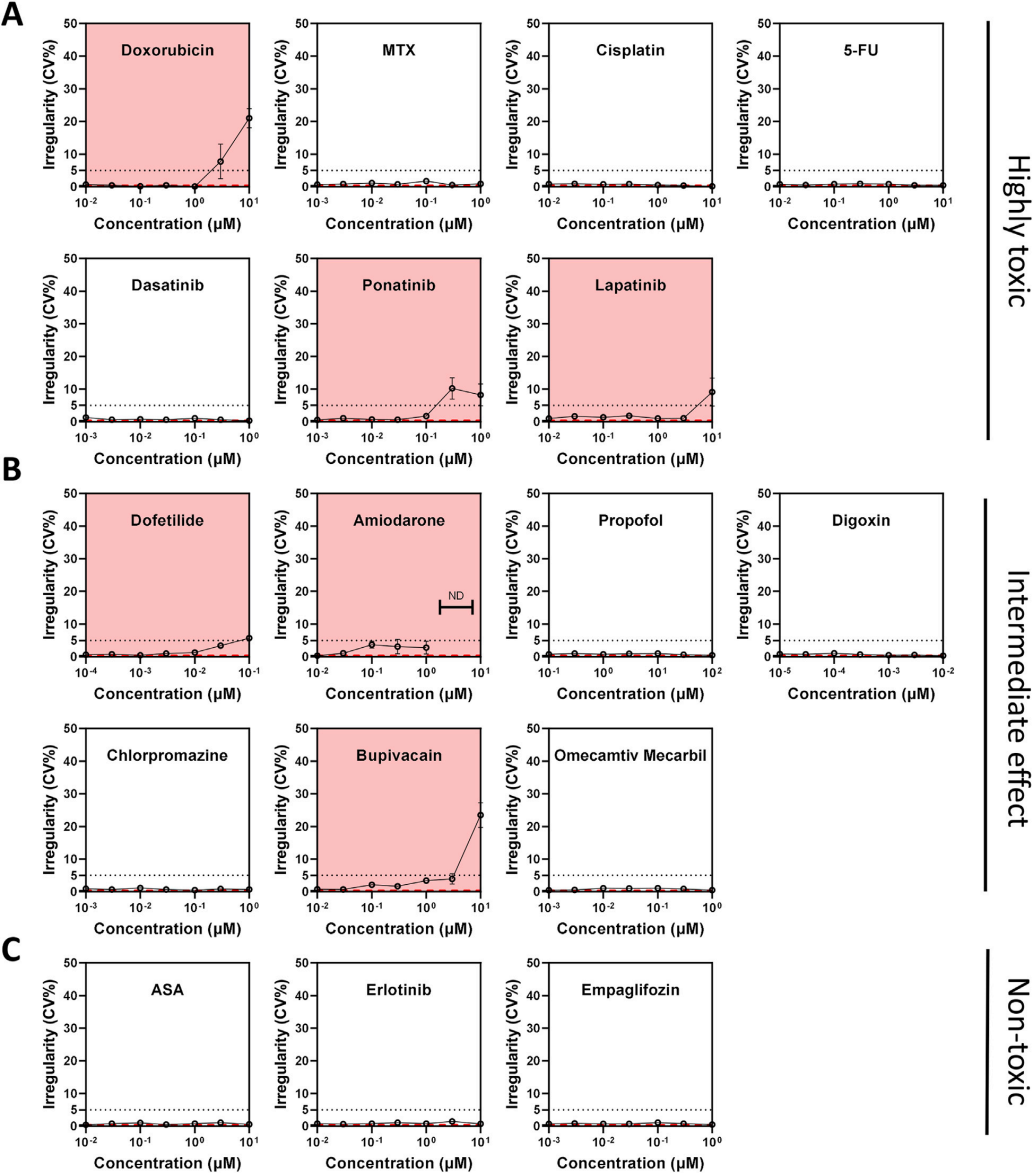
Compound-induced changes in beat rate irregularity (BRI) across toxicity classes in hiPSC-CMs. **(A–C)** Beat rate irregularity (BRI) assessed by multi-electrode array (MEA) field potential recordings after 24-h compound treatment. BRI >5% (dotted line) was used as a threshold to indicate arrhythmic activity. **(A)** Compounds classified as highly cardiotoxic. **(B)** Compounds with intermediate or unclear cardiotoxicity profiles. **(C)** Compounds considered non-cardiotoxic. The red dashed line indicates vehicle control values. Compounds highlighted with a red background indicate elevated BRI or electrical quiescence.

MEA experiments using Ncyte CM1 cells, showed doxorubicin compromised electrical activity by increasing BPM and BRI, even at sub-clinical concentrations ≥100 nM (Figure 5A; Supplementary Table S4). Findings from electrophysiology confirmed the highly cardiotoxic potential of doxorubicin, while imaging analysis additionally highlighted the cellular mechanisms involved.

**FIGURE 6:**
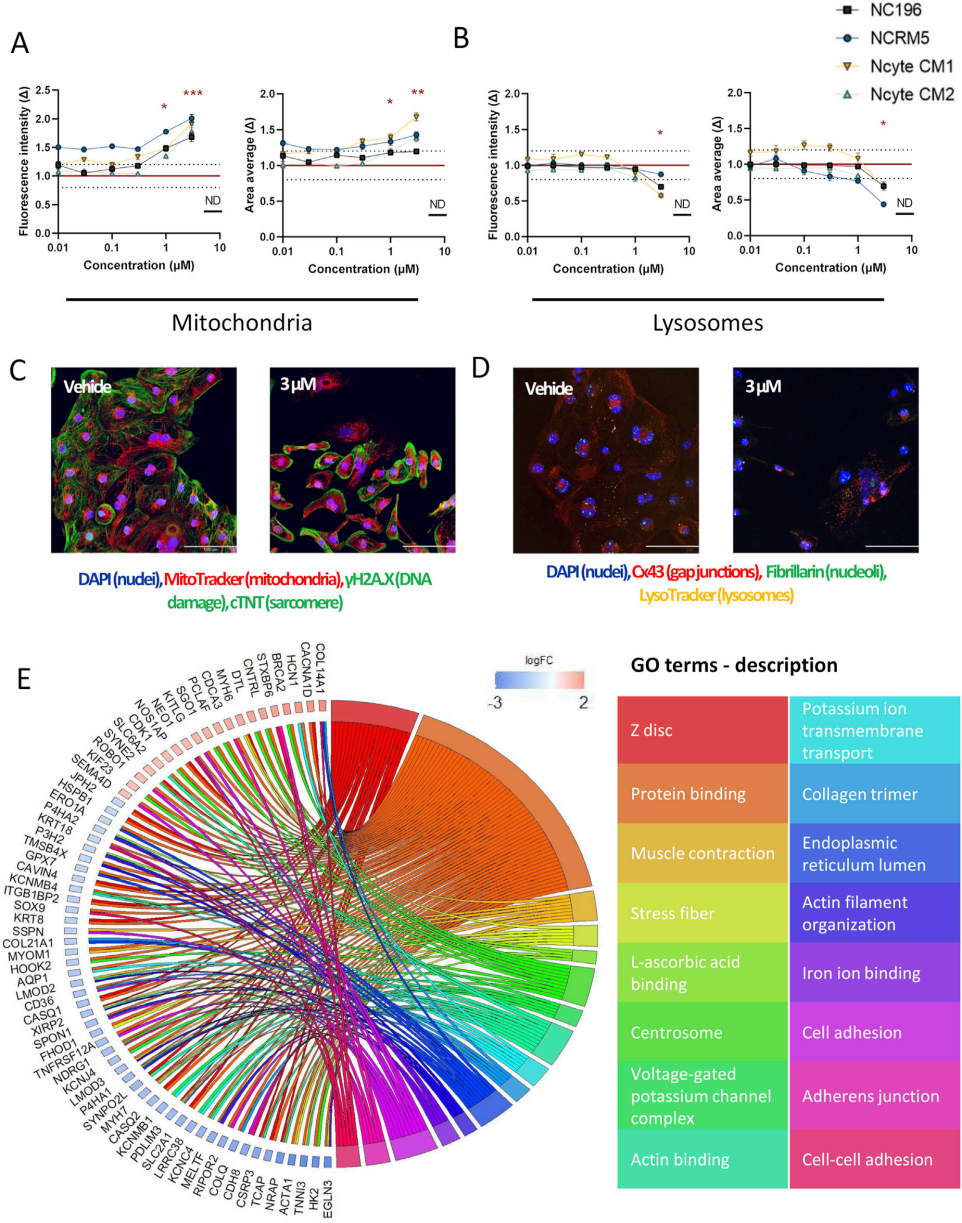
High-content imaging and transcriptomic profiling of amiodarone-treated hiPSC-CMs. **(A,B)** Morphological alterations induced by amiodarone across hiPSC-CM lines, shown as fold change (Δ) relative to vehicle control across a 7-point semi-logarithmic concentration range. **(A)** Mitochondrial signal. **(B)** Lysosomal signal. **(C,D)** Representative 40× fluorescence images of **(C)** staining for DNA damage (within the nuclei), troponin (cytoplasm), mitochondria and nuclei and **(D)** lysosomes, gap junctions, nucleoli and nuclei after 24-h incubation with 3 µM amiodarone or DMSO vehicle. Scale bar = 100 µm. **(E)** Gene Ontology (GO) enrichment analysis of RNA-seq data from hiPSC-CMs treated with 1 µM amiodarone. Displayed are non-redundant GO terms (biological process, cellular component, molecular function) with at least four differentially expressed genes and genes involved in two or more categories, along with corresponding logFC2 (effect size) values for included genes. Data are presented as mean ± SEM. High-content imaging: N = 4 (biological replicates from different hiPSC-CM lines), n ≥ 6 (technical replicates per condition). RNA-seq: N = 3 (Ncyte CM1). Statistical significance assessed by Kruskal–Wallis test; p < 0.05 values are shown (*p < 0.05,** p < 0.01, *** p < 0.001, **** p < 0.0001).

An interesting compound from the intermediate toxicity group, amiodarone, also displayed a distinct morphological phenotype. At the highest concentration tested, the detachment of hiPSC-CMs in culture rendered the collection of adequate imaging data impossible. Similarly, cellular loss has been shown in multiple cell types (Bargout et al., 2000; Balasubramanian et al., 2019). Even in the absence of major hiPSC-CM loss at 1 μM, deregulation in genes involved in cellular adhesion and cell-cell adhesion (e.g., *COL14A1↑*; *KITLG↑*, *SOX9↓*, *ACTC1↓*, *NDRG1↓*, *KCNJ4 ↓*, Figure 6E) was detected.

As a cationic amphiphilic drug, amiodarone promotes the buildup of phospholipids within the endosomal-lysosomal system i.e. phospholipidosis (Buratta et al., 2015; Niimi et al., 2016; Sagini et al., 2021). In accordance with effects observed in primary human cardiomyocytes (Krajcova et al., 2023), increased mitochondrial fluorescence intensity and detected signal area (up to 1.8 ± 0.1 times and 1.7 ± 0.6 times), and lysotoxic effects with decreased fluorescence intensity (up to 0.69 ± 0.1 times) were observed in our study (Figures 6A–D). Additionally, downregulation of genes linked to lysosomal and mitochondrial function, such as *P4HA1,* were detected. Notably, this gene is also associated with the endoplasmic reticulum lumen cellular component, genes of which (*P4HA1*, *SPON1*, *P3H2*, *COL21A1*, *GPX7*, *P4HA2*, *MELTF*, *ERO1A*) were also downregulated (Figure 6E).

In MEA analysis, amiodarone slowed down BR at ≥0.3 µM, without causing FPD prolongation (Supplementary Table S4). Cessation of beating was detected at concentrations ≥3 µM. This aligns with the findings of the CiPA studies (Blinova et al., 2017; Blinova et al., 2018; Blinova et al., 2019). Moreover, treatment also affected processes regulating ion channels (e.g., *KCNJ4↓*, *HCN1↑*, *CASQ2↓*), muscle contraction, and actin filament organization (Figure 6E).

Out of the seventeen compounds tested, ten exhibited effects on any electrophysiology parameters at varying concentrations (Figure 5; Supplementary Table S4). Specifically, seven compounds—doxorubicin, amiodarone, lapatinib, bupivacaine, erlotinib, ponatinib, and dofetilide—were associated with either arrhythmia and/or quiescence (Figure 5). Digoxin shortened the FPD whereas dofetilide and cisplatin caused prolongation. Interestingly, erlotinib displayed a bell-shaped dose-response curve demonstrating complex interactions between dose and target engagement. Chlorpromazine had a mild effect on FPD; however, this effect size was not dependent on concentration. Six compounds had no effect on electrophysiology, including two non-toxic compounds (empagliflozin, aspirin), methotrexate, 5-fluorouracil, propofol, and omecamtiv mecarbil (Supplementary Table S4).

When examining individual morphological parameters, lysosomes were affected by doxorubicin, amiodarone, ponatinib, erlotinib, and lapatinib. Lapatinib also decreased peroxisomal area (Supplementary Figure S6). DNA damage was observed with doxorubicin only, a compound that also impacted nucleoli and CX43 (Figure 4). Mitochondria were most disturbed by amiodarone (Figure 6). Sarcomere alterations (i.e., sarcomere width) were seen with doxorubicin, amiodarone, bupivacaine and dofetilide (Supplementary Figure S6).

Results confirm that morphological analysis detects diverse mechanisms of potential cardiotoxicity and highlight its advantages as a complementary method for providing a more comprehensive cardiotoxicity assessment.

### 3.3 Clustering analysis delineates cardiotoxic drug profiles by effect size and mechanism of action

Morphological profiling generates extensive datasets, in this study comprising of 205 parameters (Supplementary Tables S5, S6), enabling the application of bioinformatic clustering techniques.

To explore broad phenotypic effects of the compounds a PCA-based approach was used. All seven concentrations tested per compound were included and treated as a single group, enabling detection of patterns emerging at any exposure level and circumventing the dose-dependent variability in compound response across hiPSC-CM lines. For each compound, a morphological ‘fingerprint’ – reflecting the pattern of morphological perturbation induced by a compound—was generated by analyzing the loading scores of the first principal component (PC1) averaged across cell lines/batches. These scores represent the relative contribution of each morphological feature to the primary axis of variance in the dataset and served as input for clustering analysis, allowing for a normalized data comparison across.

**FIGURE 7:**
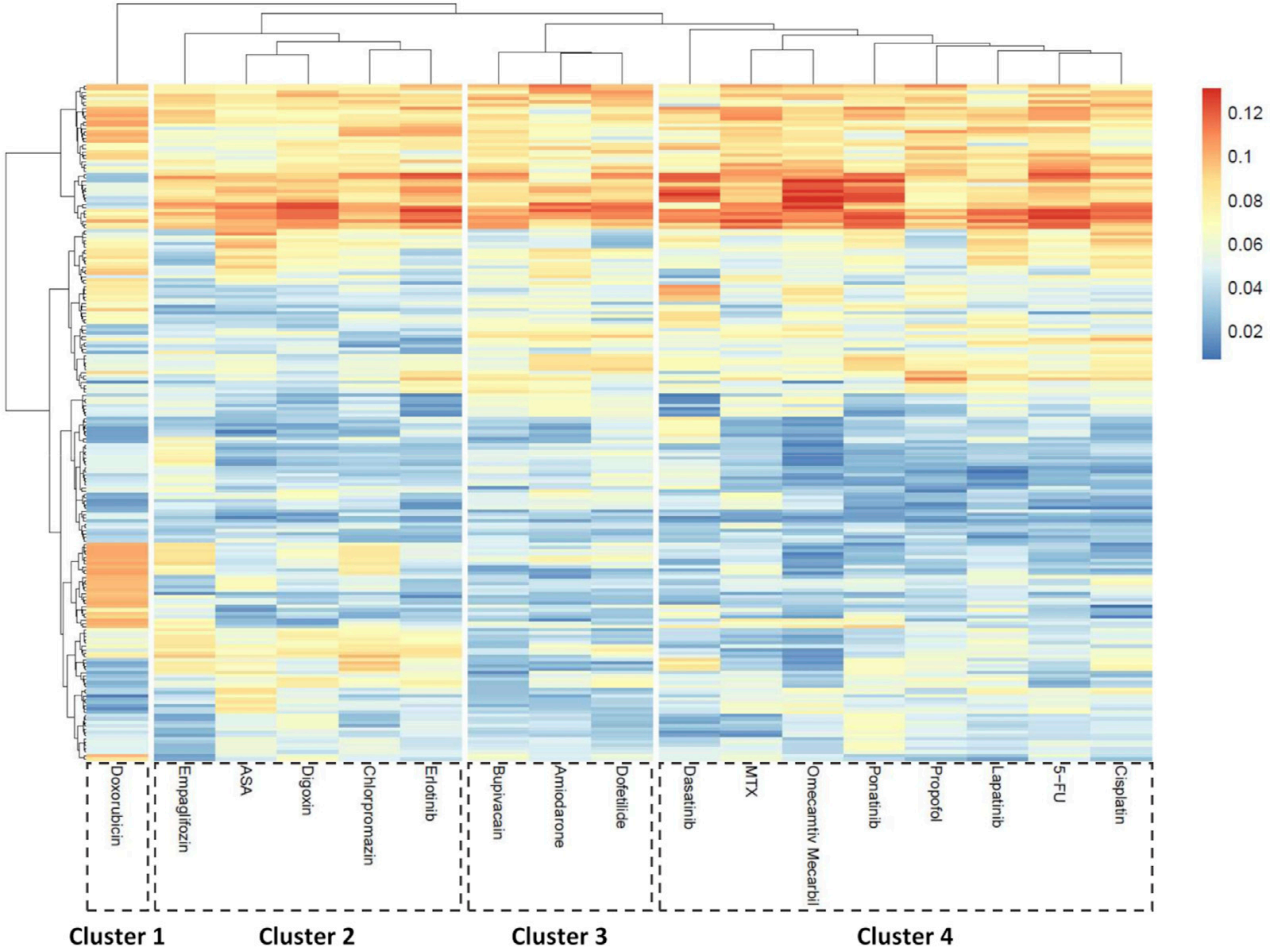
Morphological feature-based clustering of compound responses in hiPSC-CMs. Principal component analysis (PCA) was performed for each compound compared to vehicle control to identify key morphological descriptors. The heatmap shows averaged absolute loading scores of the first component (PC1) per parameter across treatments. K-means clustering was applied to the loading profiles to group compounds based on morphological similarity. N = 4 (biological replicates from different hiPSC-CM lines), n ≥ 6 (technical replicates per condition).

Using this approach, drugs could be divided into 4 clusters (Figure 7). Doxorubicin, as expected, formed its own cluster (cluster 1) characterized by prominent changes to the nuclei and cytoplasm. Interestingly, known non-cardiotoxic compounds (erlotinib, aspirin, and empagliflozin) clustered together with chlorpromazine, an antipsychotic agent and digoxin, a Na/K ATPase inhibitor (cluster 2). A smaller cluster (cluster 3) included ion-channel blockers, amiodarone, dofetilide and bupivacaine. This cluster represents compounds that affect cardiac electrophysiology (Falk and Decara, 2000; Lim et al., 2006; Stoetzer et al., 2016; Vaiciuleviciute et al., 2021; Tagle-Cornell et al., 2024). Evidently, bupivacaine, as observed in the clinic (Cotileas et al., 2000; Osama et al., 2024), led to decreased beat rate (3, 10 µM) (Supplementary Table S4) and increased irregularity (10 µM) (Figure 5). The fourth cluster (cluster 4) was characterized by mixed toxicity on multiple organelles, such as changes in gap junctions. The increase in cytosolic connexin CX43 observed in our study for both ponatinib and doxorubicin is in line with literature reports (Pecoraro et al., 2015; Pecoraro et al., 2017; Madonna et al., 2021) and may relate to heterogeneous post-treatment redistribution (Fontes et al., 2012). Notably, lapatinib was the only compound that changed peroxisomal parameters (Figure 7; Supplementary Figure S6). Correspondingly, GO term analysis showed deregulation of fatty-acid metabolic processes as well as peroxisomal β-oxidation including genes such as *ACSL1*, *ACSL3*, and *ACOX1* (Supplementary Table S7).

In addition to unsupervised clustering, supervised classification was used to further investigate whether compound-induced phenotypes could be aligned with known clinical cardiotoxicity profiles. To this end, sPLSDA (Perez-Enciso and Tenenhaus, 2003) was applied to replicate-level data to assess the relationship between morphological and electrophysiological alterations and FDA label-based toxicity classifications (Supplementary Table S8). One concentration per compound was selected for this analysis, following the same rationale as for RNA-seq experiments: the dose that elicited the most pronounced functional effect in MEA recordings without significant loss of viability (Table 1).

**FIGURE 8:**
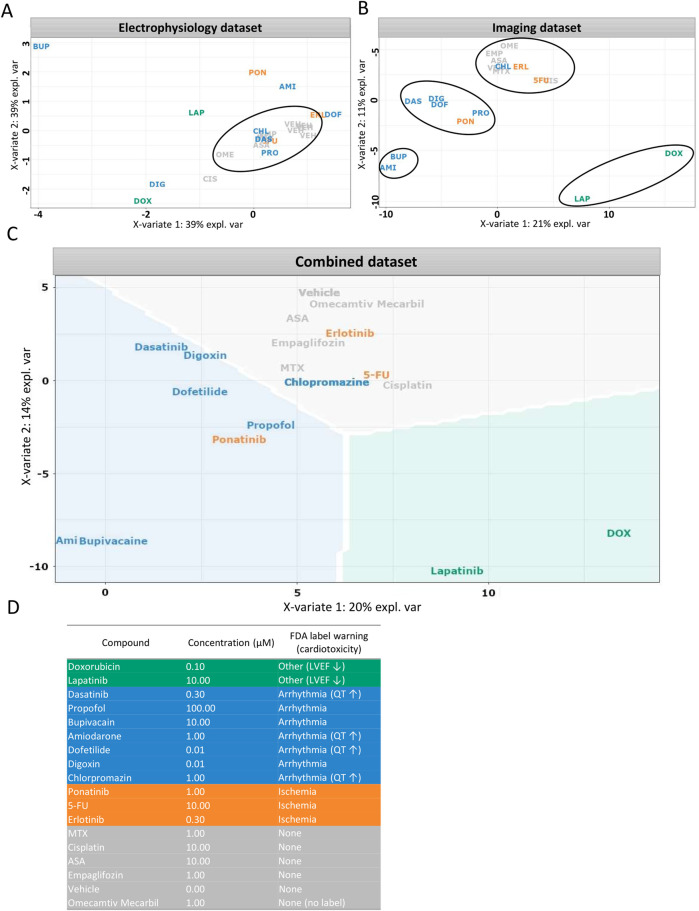
Supervised clustering based on clinical side effects. **(A–C)** Partial least squares discriminant analysis (sPLS-DA) was used to visualize compound clustering based on different datasets: **(A)** electrophysiological features, **(B)** imaging-derived morphological features, **(C)** combined dataset weighted by data type. **(D)** Compound toxicity groups were defined according to FDA label warnings. Each data point represents the vehicle-normalized median feature value for a compound at one selected concentration per assay. Data is shown for Ncyte CM1 only.

Lapatinib and doxorubicin, known for their potential effect on left ventricular ejection fraction at least in some patients (Chatterjee et al., 2010; Hsu et al., 2018; Waliany et al., 2023), visibly clustered together when morphological and electrophysiological parameters were combined (Figure 8). However, this similarity was not evident from electrophysiology parameters alone. As a separate example, in the electrophysiology dataset TKIs; erlotinib and ponatinib showed similarity to dofetilide and to amiodarone, respectively. Both are known clinically to be associated with ischemia (Gover-Proaktor et al., 2017; Haguet et al., 2020) yet clustered with arrhythmogenic- and non-toxic compounds, respectively, in the imaging dataset. Unlike other compounds in the ischemia group, ponatinib carries an FDA black box warning for arrhythmia risk, (Vassallo and Trohman, 2007), which could explain its localization alongside the arrhythmia group. From the arrhythmia group, chlorpromazine showed no separation from the non-cardiotoxic compounds in either imaging alone, electrophysiology assays alone, or combined clustering. Similarly, other studies also indicate little effects of chlorpromazine in electrophysiology (Blinova et al., 2017; Blinova et al., 2018; Blinova et al., 2019; Yu et al., 2019). In summary, the addition of functional data to morphology, increased the separation of the clusters formed based on clinical information resulting in 76% of compounds clustered according to their known clinical toxicity (Figure 8C). Misclassified compounds were primarily from the ischemia group. Clustering based on imaging-derived morphological features alone (Figure 8B) produced compound groupings that closely matched those observed in the combined dataset. This convergence suggests that high-content imaging captures phenotypic signatures that are strongly aligned with clinical toxicity classifications, and in some cases may be sufficient for compound-level discrimination without additional functional data. The similarity between these panels underscores the value of imaging as a robust and scalable standalone modality for cardiotoxicity screening, particularly in settings lacking access to electrophysiological platforms. However, for certain compounds with specific functional effects (e.g., ion-channel blockers), electrophysiology provides unique complementary insight (Figure 8A).

## 4 Discussion

Utilizing hiPSC-CMs treated with a panel of reference compounds, our approach integrates traditional electrophysiology with morphological profiling to establish a more comprehensive and innovative *in vitro* assessment of cardiac safety. Our findings demonstrate that while drug classification is possible using functional readouts alone, combining it with morphological assays in a multiplexed approach provides a more robust framework for cardiac-safety assessment. Imaging not only complements electrophysiology but may have the potential to replace it in certain contexts, as it offers mechanistic insights unattainable *via* electrophysiology alone and is a technology suitable for labs lacking dedicated expertise and equipment for electrophysiology assays.

Assessing cardiac safety *in vitro* remains a challenge. Early approaches focused on the electrophysiological characteristics of hiPSC-CMs; linking corrected FPD and presence of arrhythmia *in vitro* to native ECG endpoints (Yamamoto et al., 2016) or calculating relative TdP scores, (Ando et al., 2017), suggesting that hiPSC-CMs are reliable models for assessing clinical proarrhythmic risk. As the field advances, in parallel to animal testing being phased out (Zushin et al., 2023), hiPSC-CMs are expected to play an increasingly important role in the evolving regulatory frameworks such as the new ICH guideline on QT/QTc evaluation (E14/S7B Q&A, adopted 2022), which explicitly acknowledge hiPSC-CM assays as a new nonclinical tool.

Beyond electrophysiology, tolerance interval calculations *via* weighted scoring matrices derived from multiple readouts (e.g., impedance, MEA, and calcium transients) enabled the consolidation of different drug effects into one hazard label (Kopljar et al., 2018). Genetic variability between individuals has been accounted for by comparing healthy and diseased hiPSC-CMs, which in combination with multiple parameters, led to the development of a “cardiac safety index” (Sharma et al., 2017).

In the present study, to circumvent bias introduced by polymorphisms or line-to-line variability, we included multiple hiPSC-CM lines from different genetic backgrounds. Moreover, hiPSC-CMs were generated *via* both 2D and 3D differentiation methods. Traditional electrophysiology readouts were combined with morphological analysis to integrate diverse aspects of cardiotoxicity into the analysis.

A novel approach to assessing compound-induced toxicity involves image-based morphological analysis that captures rich subcellular information as a set of features describing drug responses (Hughes et al., 2020; Nyffeler et al., 2020; Way et al., 2021). The JUMP- (Cimini et al., 2023) and Oasis Cell Painting Consortium are recent initiatives for enhancing morphological screening by standardizing the traditional Cell Painting assay (Bray et al., 2016; Pahl and Sievers, 2019). However, at the moment, they are limited in terms of concentration range and cellular models utilized (Chandrasekaran et al., 2024).

In contrast, our study extends these efforts by applying morphological profiling to hiPSC-CMs across a broader, clinically relevant concentration range. The panel of subcellular markers was also expanded compared to traditional Cell Painting to capture a more extensive set of organelle-specific responses. Morphological profiling not only identified potential cardiotoxicity, but also enabled a mechanistic grouping of compounds. Together with MEA, these two modalities provide both a global, dose-inclusive view of morphological response (PCA) and a more detailed classification of compound effects (sPLS-DA) linked to clinically observed toxicities, revealing distinct aspects of drug-induced cardiotoxic effects and highlighting the need for more holistic approaches in pharmaceutical screening.

Assessing doxorubicin’s toxicity profile served as proof of concept for further analysis. Clinical cardiotoxic side-effects of doxorubicin range from mild paroxysmal non-sustained arrhythmias to complex, irreversible and fatal structural alterations of the myocardium, the risk of which tends to increase in a dose-dependent manner (Chatterjee et al., 2010). Preclinical studies have confirmed that doxorubicin enacts its cytotoxic role through reactive oxygen species formation, damage to the DNA, and alterations of calcium and mitochondrial homeostasis (Parra et al., 2008; Carvalho et al., 2014; Adamcova et al., 2019; Osataphan et al., 2020; Wu et al., 2022). Doxorubicin acts primarily *via* inhibition of topoisomerase IIα causing DSBs (Tewey et al., 1984). As adult cardiomyocytes tend to lack the IIα isoform, (Turley et al., 1997), whereas it is relatively prevalent in hiPSC-CMs (Cui et al., 2019), hiPSC-CMs might be more suitable for detecting doxorubicin toxicity. Morphological analysis repeatedly confirmed doxorubicin’s effect on inducing DSBs, however, contrary to literature, mitochondrial damage (Osataphan et al., 2020; Wu et al., 2022) and fragmentation (Parra et al., 2008) could not be observed in this study. Sensitivity to doxorubicin varies per hiPSC line and heavily depends on incubation time (Louisse et al., 2017; Adamcova et al., 2019). Longer incubation times, repeated dosing, or use of alternative mitochondrial dyes could enable the detection of these changes. Yet, RNA-seq data pointed towards an effect on mitochondria as genes such as *NDUFB3* (part of complex I), *CYCS* (key player in the electron transport chain and apoptosis), and *SLC25A5* were upregulated, similar to differential expression patterns found in literature (Holmgren et al., 2018). Interestingly, doxorubicin significantly increased the LysoTracker signal. We hypothesize that this effect could be explained by the presence of non-degraded autolysosomes. Doxorubicin’s role in autophagy has been debated; nonetheless, there is evidence pointing towards blocking autophagic flux in animal- and cell-models in a process which is associated with a significant buildup of non-degraded autolysosomes (Li et al., 2016).

Another compound that exemplifies the advantages of a multiparametric approach is amiodarone, a potent antiarrhythmic, considered to be one of the most effective for treatment of ventricular arrhythmia and atrial fibrillation (Vassallo and Trohman, 2007). Amiodarone reduced BPM at concentrations below 0.3 µM without FPD prolongation during acute treatment, despite the QT warning present on its FDA label (Lim et al., 2006). Lack of sensitivity to the compound–which was also evident in other studies (Blinova et al., 2017) – could potentially be attributed to alterations in relative expression of individual ion channels compared to adult cardiomyocytes (Schmid et al., 2021).

A contrasting example is erlotinib, a TKI rarely associated with cardiac side-effects (Sayegh et al., 2023). In this study erlotinib induced detectable electrophysiological changes in MEA assays; however, morphological profiling alone and in combination with electrophysiology it clustered with non-toxic compounds. Since MEA measurements were conducted only in the Ncyte CM line, one possible explanation is individual donor-specific genetic variation influencing drug response. Notably, although rare, clinical reports do document cases of QT prolongation associated with erlotinib treatment (Kloth et al., 2015). These findings suggest that in some instances, electrophysiological assays may detect functional liabilities that are not yet evident on the morphological level.

A broader comparison across tyrosine kinase inhibitors (TKIs) further illustrates the assay-specific nature of detection. Other TKIs in this study—lapatinib, dasatinib, and ponatinib—showed divergent profiles. Lapatinib and ponatinib both affected morphological and electrophysiological parameters and clustered separately from low-risk compounds. This is consistent with their known clinical cardiotoxicity, including reports of QT prolongation and, in the case of ponatinib, an FDA black box warning for severe cardiovascular events (Vassallo and Trohman, 2007; Coppola et al., 2018; Ando et al., 2020). In contrast, dasatinib did not significantly affect MEA parameters, despite clinical reports of cardiotoxicity. This divergence highlights how compound-specific mechanisms and assay limitations can influence the detectability of adverse effects.

Extending the scope to the complete compound library, we were able to show that the *in-vitro* imaging data derived from 205 cellular parameters correlate well with clinically-observed side effects (Figure 8). FDA labels were systematically reviewed and categorized into major cardiovascular risk groups: arrhythmia, ischemia, structural abnormalities (e.g., reduced ejection fraction or cardiomyopathy), and none. Clustering based on imaging alone captured these groupings to a large extent, and inclusion of electrophysiology data further improved separation. One exception was the ischemia group, which did not resolve into a distinct cluster. This may reflect the fact that ischemic cardiotoxicity often involves endothelial dysfunction in addition to direct cardiomyocyte effects (Soultati et al., 2012; Polk et al., 2014; Gover-Proaktor et al., 2017; Haguet et al., 2020) a mechanism not captured by our hiPSC-CM model.

Despite the promising outcomes, several limitations must be acknowledged. One primary constraint is the relative immaturity of hiPSC-CMs, which may not fully recapitulate physiological responses of native cardiomyocytes. Lack of co-culture systems to mimic the complex interactions between different cell types and accounting for systemic effects of compounds is another significant limitation. Translatability across larger populations may be hindered by the relatively small number of hiPSC lines included. The range of concentrations per compound tested limited the size of the compound library which may have constrained the power of clustering drug mechanisms. Finally, specificity issues related to dyes and the high costs of antibodies used in the study also present challenges.

Nonetheless, our combinatorial protocol is valuable for developing more predictive *in vitro* cardiotoxicity assessment models. Therefore, we propose integrating morphological readouts into future cardiac safety-indices as we have shown that certain types of compound induced changes may be overlooked by traditional safety assays.

High-content imaging, in combination with morphological profiling is a powerful tool to assess compound effects *in vitro*. Staining protocols were optimized for morphological profiling of organelles and used to screen a diverse library of compounds. Organelle profiling revealed concentration- and compound-dependent effects that traditional readouts only, such as electrophysiology, might not detect. This highlights the added value of combined multi-assay analytics. In addition, many of the parameters specific to each compound were corroborated by alterations in gene-expression levels, supporting that multi-assay analysis can elucidate mechanistic insights into compound effects.

Ultimately, a parallel can be drawn between clinical profiles of toxicity and *in-vitro* findings in hiPSC-CMs, making the platform appropriate for pre-clinical cardiac safety screening of new compounds, and enabling safer medicines to reach the clinic.

## Data Availability

The datasets presented in this study can be found in online repositories. The names of the repository/repositories and accession number(s) can be found below: https://www.ncbi.nlm.nih.gov/geo/, GSE289264.
