# Supplementary figures and images for: Integral approach to organelle profiling in human iPSC-derived cardiomyocytes enhances in vitro cardiac safety classification of known cardiotoxic compounds
Source: Front Toxicol. 2025 Aug 21;7:1644119. doi: 10.3389/ftox.2025.1644119 (PMC12408628; doi:10.3389/ftox.2025.1644119)

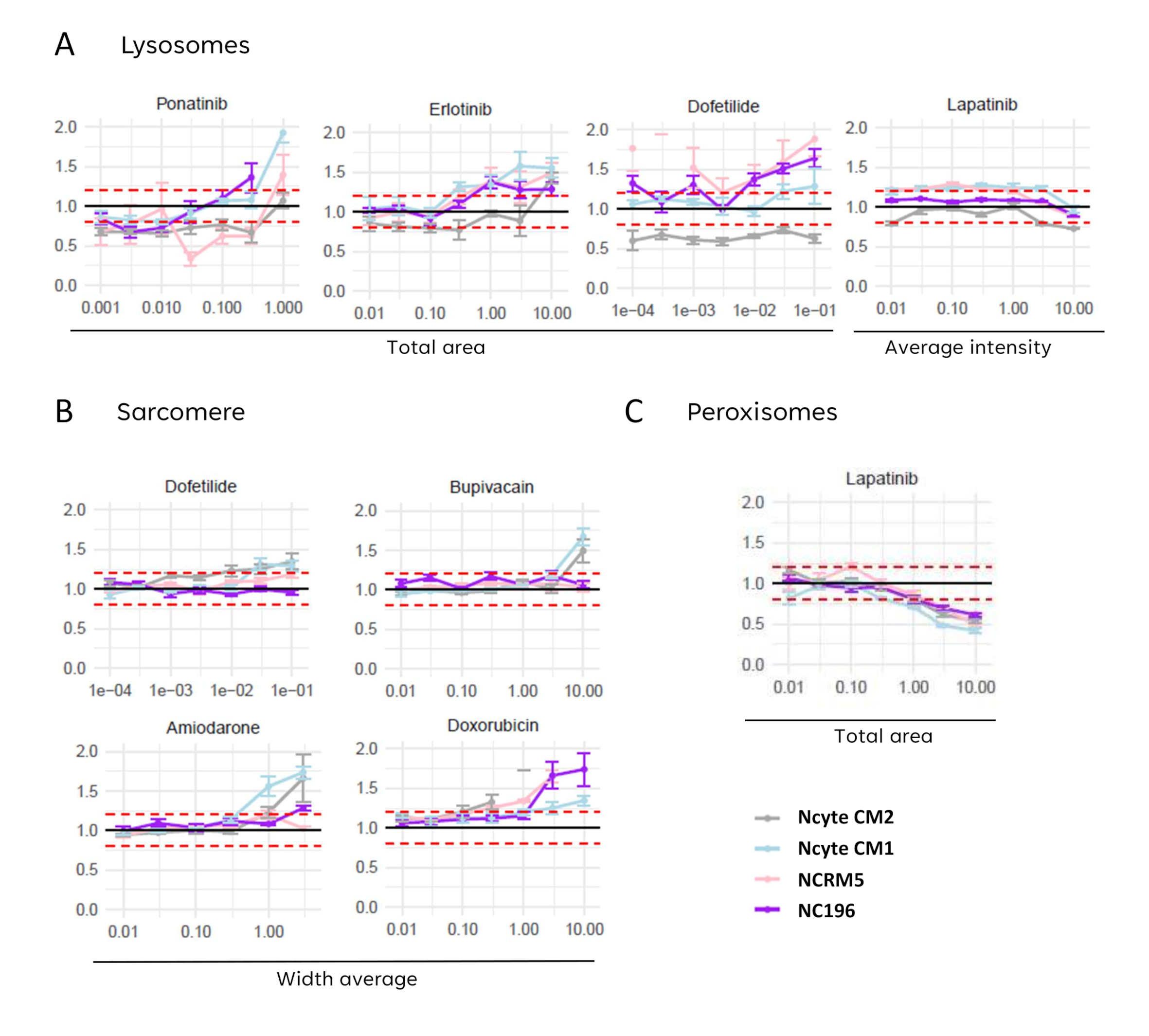

Supplement: Supplementary file 1 [file Image6.tif]

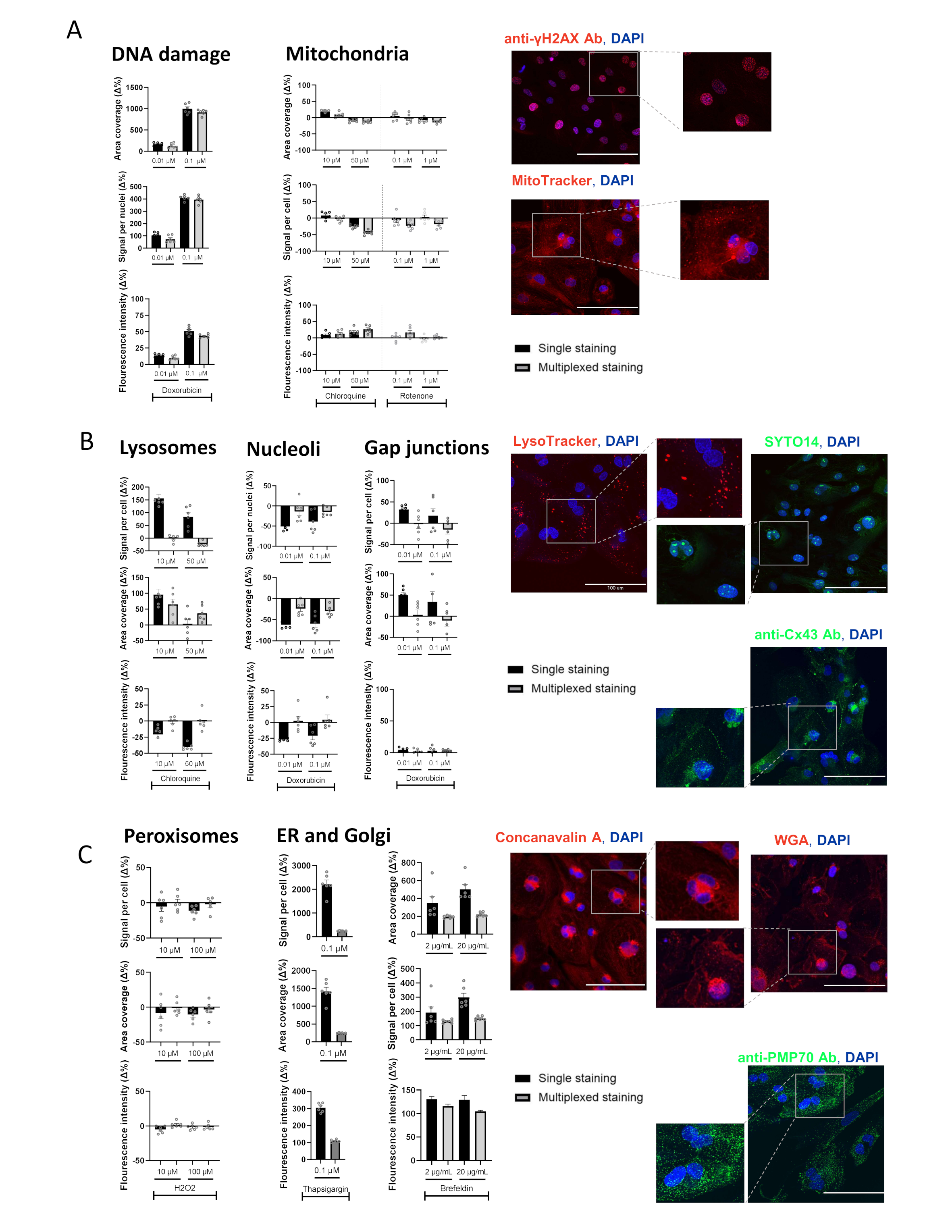

Supplement: Supplementary file 3 [file Image3.tif]

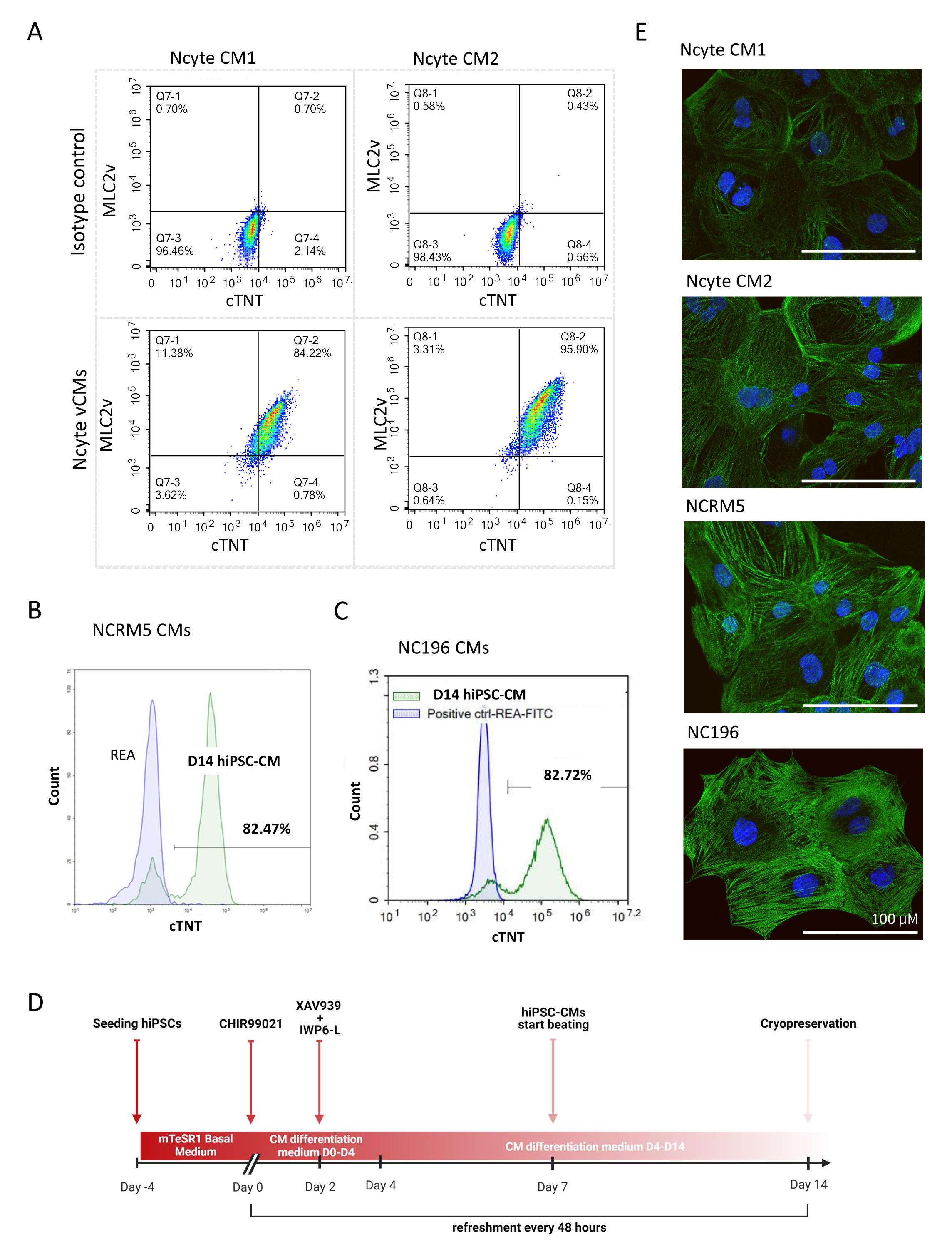

Supplement: Supplementary file 4 [file Image4.tif]

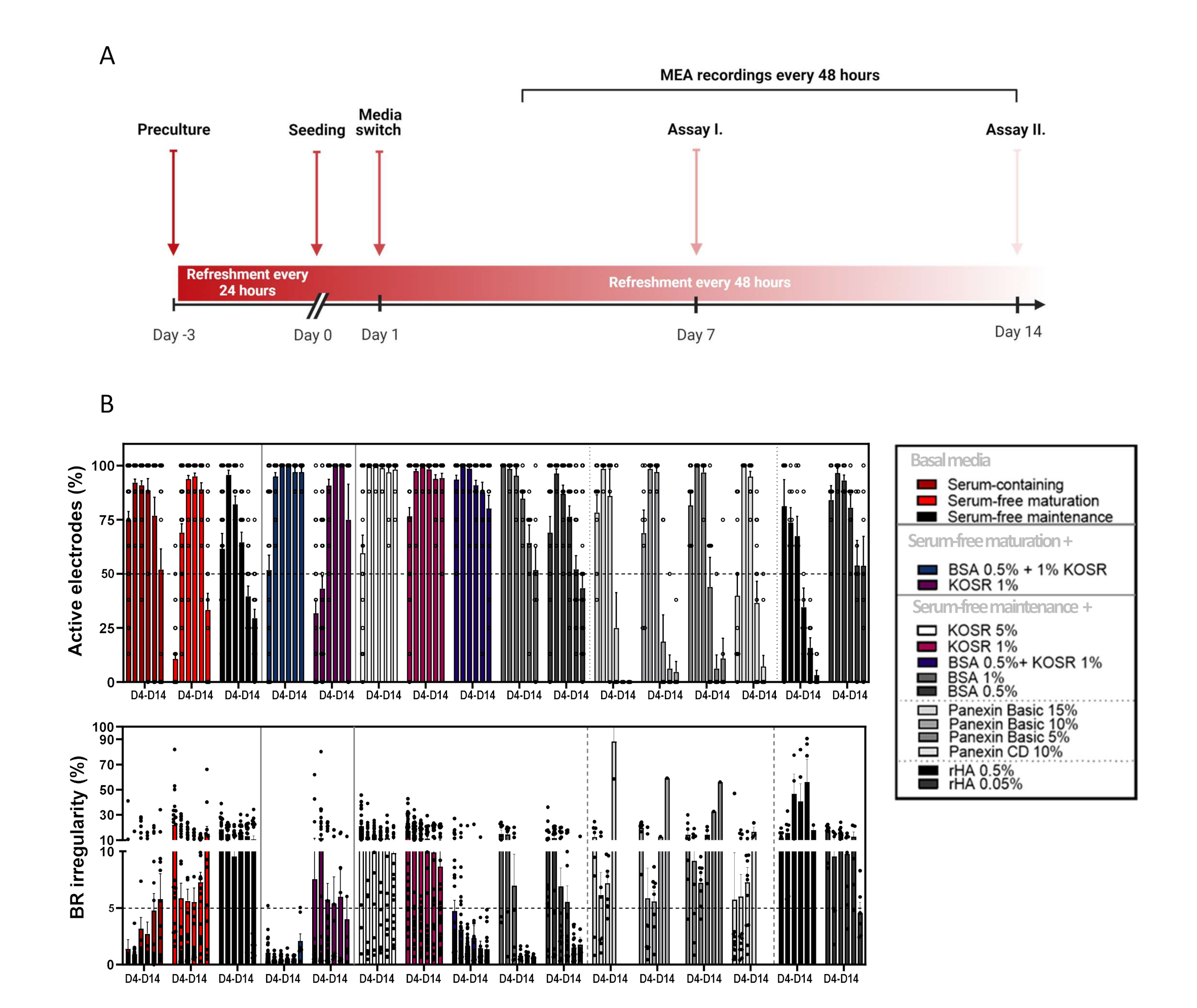

Supplement: Supplementary file 5 [file Image2.tif]

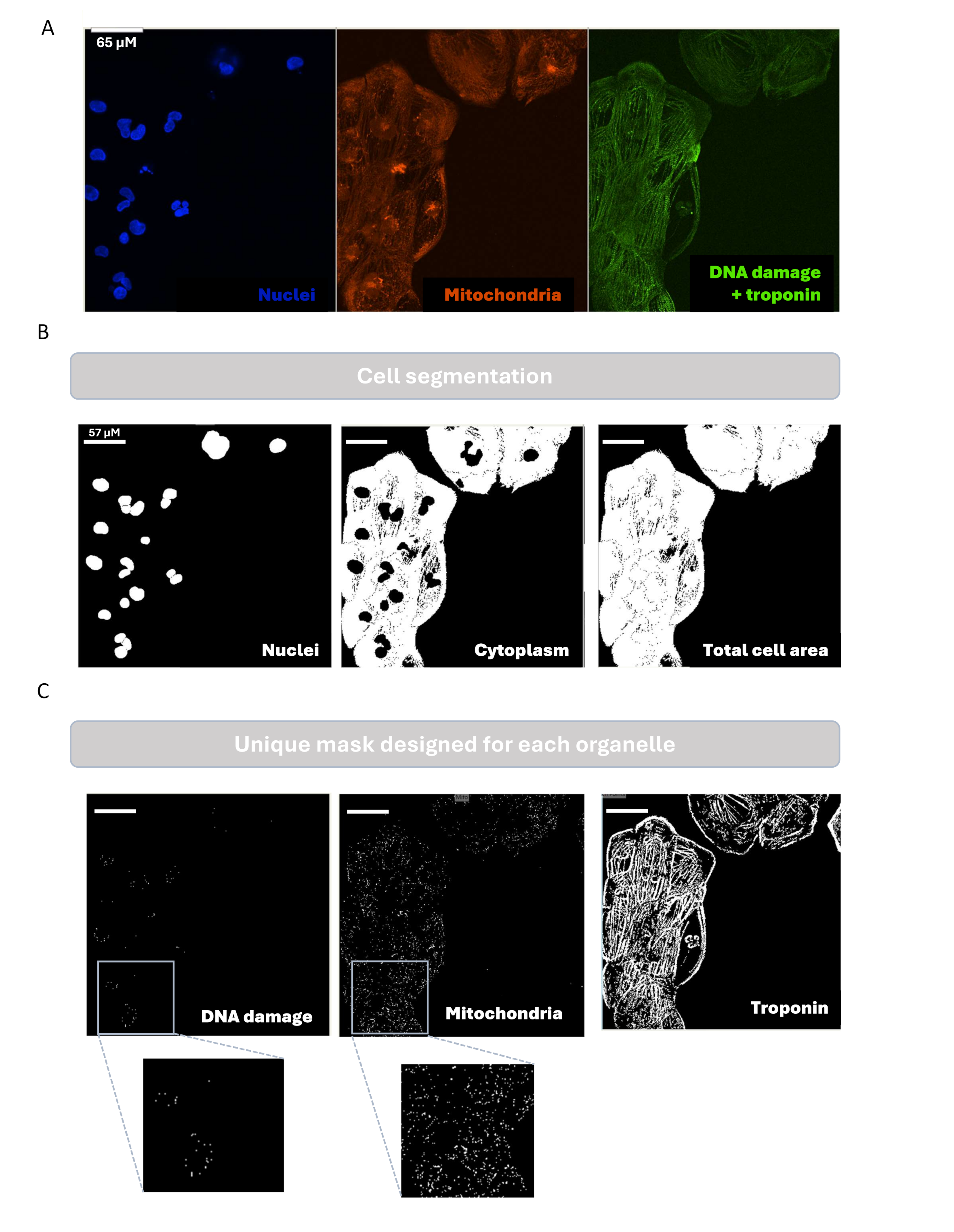

Supplement: Supplementary file 6 [file Image1.tif]

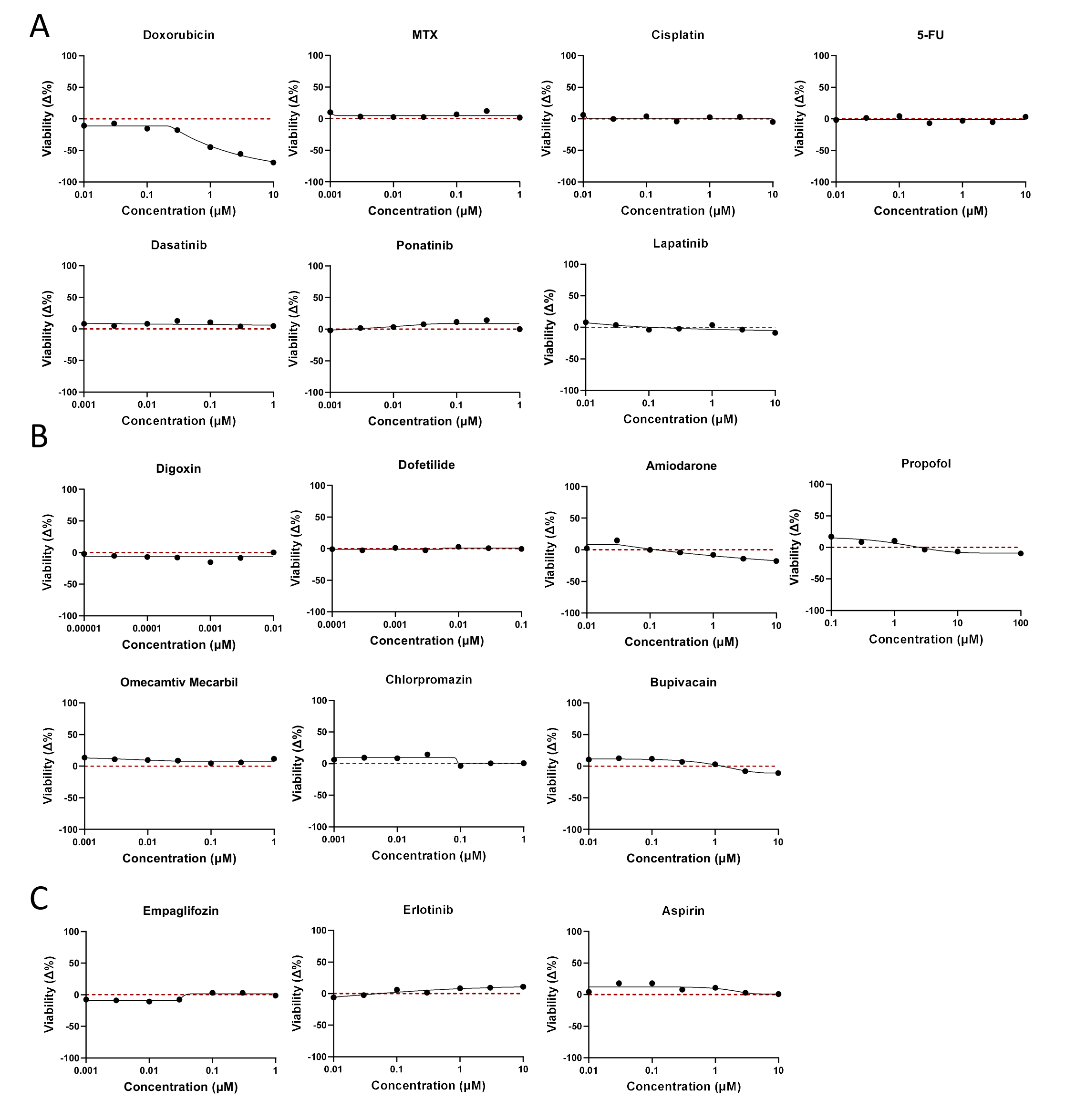

Supplement: Supplementary file 7 [file Image5.tif]
