# Supplementary material for: Integral approach to organelle profiling in human iPSC-derived cardiomyocytes enhances in vitro cardiac safety classification of known cardiotoxic compounds
Source: Front Toxicol. 2025 Aug 21;7:1644119. doi: 10.3389/ftox.2025.1644119 (PMC12408628; doi:10.3389/ftox.2025.1644119)
Supplement: Supplementary file 2 [file Supplementaryfile1.docx]

Supplementary Material

# Supplementary Methods

## Human induced pluripotent stem cell (hiPSC) reprogramming

For generation of the NC196 hiPSC line, polymorphonuclear blood cells (PBMCs) were obtained from the blood of healthy donors following informed consent and appropriate ethical approval by METC azM/UM (2019-1238-A-15), at Maastricht University. PBMC were isolated using Ficoll Histopaque (Sigma Aldrich) and expanded for 9 days in StemSpan™ SFEM II (StemCell Technologies) supplemented with factors promoting cell growth (SCF, IGF1, EPO, IL3 and dexamethasone). Erythroid progenitor cells (EPCs) were reprogrammed using electroporation with Epi5™ Episomal hiPSC Reprogramming Kit (Invitrogen). Modified cells were plated onto culture dishes covered with hESC-qualified Matrigel® Matrix (Corning Life Sciences) and maintained in Reprogramming Medium ReproTeSR (Stem Cell Technologies) until the appearance of hiPSC colonies, which were picked manually and expanded in mTeSR™1 (Stem Cell Technologies).

As hiPSC were reprogrammed using non-replicating EBNA-based episomal vectors, clones after 10 passages were tested for the elimination of the EBNA virus using qPCR. Total genomic DNA was extracted using innuPREP DNA Mini Kit 2.0. As a positive control, gDNA isolated on day 1 after reprogramming was used. qPCR was performed using primers; pEP4-SF2-OriP:5’-ATCGTCAAAGCTGCACACAG-3’ and EBNA-REV:5’-GGTTTTGAAGGATGCGATTAAG-3’ according to the manufacturer's instructions of iQ™ SYBR® Green Supermix (Bio-Rad).

## hiPSC culture

hiPSCs were maintained at 37°C and 5% CO2, in mTeSR™1 Basal Medium (StemCell Technologies) on Matrigel (BD Biosciences) coated tissue culture plates according to the supplier’s protocol. hiPSC cultures were routinely tested for mycoplasma using MycoAlert kit (Lonza).

## Cardiac differentiation of hiPSCs in 2D monolayer

hiPSCs were maintained for at least 4 passages after thawing, before cardiac differentiation. Cardiac differentiation was induced in a monolayer culture using timed addition of Ncardia’s proprietary media compositions for cardiomyocyte differentiation. Medium was refreshed every 48h. Contracting cells were observed from day 7 and onwards. Cultures were monitored and maintained until day 14. On day 14, hiPSC-CMs were dissociated using 1X TrypLE™ Select Enzyme (Gibco™) for 10 minutes at 37°C, cryopreserved using Ncardia’s proprietary cryopreservation medium, and stored in liquid nitrogen.

## Flow cytometry to confirm pluripotency and assess cardiac-differentiation efficiency

hiPSCs were dissociated with Accutase® (Invitrogen™) solution at 3 passages after thawing. hiPSC-CMs were dissociated using 1X TrypLE™ Select Enzyme (Gibco™). Samples were fixed and permeabilized with Inside Stain Kit (Miltenyi Biotec) according to manufacturer’s instructions. Dissociated hiPSCs were stained for assessment of pluripotency markers – recombinant IgG1 to human SOX2 conjugated to fluorescein isothiocyante (FITC) (Miltenyi Biotec, 130-120-721); human recombinant IgG1 to human OCT3/4 isoform A conjugated to allophycocyanin (APC) (Miltenyi Biotec, 130-117-709); and human recombinant IgG1 to human NANOG conjugated to APC (Miltenyi Biotec, 130-120-704). Dissociated hiPSC-CMs were stained – for cardiac troponin T (TNNT2) – recombinant human IgG1 to human TNNT2 (Miltenyi Biotec, 130-119-575) –. Isotype controls – FITC-conjugated recombinant human IgG1 (Miltenyi Biotec, 130-118-354) and APC-conjugated recombinant (Miltenyi Biotec, 130-120-709) – were used to establish gating. Samples were analyzed using a ACEA NovoCyte® 2000 Flow Cytometer (Agilent) instrument. Data was analyzed using the NovoExpress Software (Agilent).

# Supplementary Figures and Tables

## Supplementary Figures

**Supplementary Figure 1.** Schematic overview of high-content imaging analysis workflow. (A) Representative images from three individual imaging channels (from staining panel 1) illustrate staining of cellular compartments (nuclei, mitochondria, DNA damage and cardiac troponin T) prior to computational analysis. (B) Image analysis involved automated cell segmentation based on cytoplasmic area (Texas Red channel) and nuclear area (DAPI) (C) followed by identification of specific organelles or targets using marker-specific fluorescence and localization (i.e. DNA damage within nuclear area).

**Supplementary Figure 2.** Evaluation of media effects on basic electrophysiology parameters in hiPSC-CMs. (A) Schematic outline of the experimental design. (B) Comparison of various serum-free media formulations including bovine serum albumin (BSA), Knock-out Serum Replacement (KOSR), recombinant human albumin (rHA), Pannexin Basic, Pannexin Chemically defined (CD) based on electrophysiological performance. Percentage of active electrodes (detection threshold >300 µV) and intra-well beat rate irregularity (>5% deviation) were used to assess spontaneous activity and rhythm stability. Media conditions were excluded if ≤ 50% electrodes were active and arrhythmic activity was higher than that observed in the serum-containing control across all time points (day 4 to day 14). Data represented as mean ± SEM, N = 3 biological replicates per condition (Ncyte CM1).

**Supplementary Figure 3.** Validation of organelle-specific staining using known positive control compounds in single staining and multiplexed protocols. (A) Staining panel I.; doxorubicin induced changes in γH2AX, and mitochondrial alterations induced by chloroquine and rotenone. (B) Staining panel II.; effects of chloroquine on lysosomes and doxorubicin induced changes in connexin 43 (Cx43), and nucleoli. (C) Staining panel III; H2O2, thapsigargin and brefeldin A was used as a positive control for peroxisomes (PMP70), endoplasmic reticuli (ER) and Golgi, respectively. For each marker, three parameters were measured: area coverage (percent of cytoplasm occupied), signal per nucleus (normalized to DAPI count), and fluorescence intensity, each expressed as Δ% relative to vehicle-treated controls. Representative fluorescence images of single stains show nuclei stained with DAPI (blue) and the corresponding organelle-specific marker. Data are presented as mean Δ% ± SEM, N = 1 biological replicate, n ≥ 6 technical replicates (Ncyte CM1). Scale bar = 100 µm.

**Supplementary Figure 4.** Characterization of cardiac identity of hiPSC-CMs included in the study. (A-C) Flow cytometry analysis of cardiac markers in hiPSC-CMs. (A) Expression of ventricular marker MLC2v and cardiac marker cTNT in bioreactor-derived Ncyte CM1 and CM2. (B, C) Expression of cTNT in NCRM5 and NC196 hiPSC-CMs, respectively, confirming cardiac identity. (D) 2D-differentiation protocol used to generate NC196 hiPSC-CMs. (E) Representative immunofluorescence images of all hiPSC-CMs stained for cTNT (green, cytoplasm) DAPI (blue) and γH2AX (green, nuclei). Scale bar = 100 µm.

**Supplementary Figure 5.** Viability assessment of hiPSC-CMs following compound treatment. (A-C) Bar graph showing DNA content as a proxy for cell viability after 24-hour treatment with compounds from the test library, expressed as percentage change to Δ% relative to vehicle-treated controls. (A) Highly toxic compounds, (B) compounds with intermediate or unknown toxicity and (C) compounds with no known cardiotoxicity. DNA content was quantified using the CyQuant fluorescent assay. Data represented as mean ± SD, N=1, n=4 (Ncyte CM1).

**Supplementary Figure 6.** Representative examples of morphological changes across hiPSC-CM lines shown as fold-change relative to vehicle controls. Compounds affecting (A) lysosomal total area and average signal intensity, (B) sarcomere width average and (C) peroxisomal signal total area. Data are presented per hiPSC-CM line/batch as mean ± SEM; n ≥ 6 (technical replicates).The bold horizontal line represents the value of no change compared to vehicle (1.0) and the dashed line the value of a 20% increase (upper dashed line) or of a 20% decrease (lower dashed line).

## Supplementary Tables

**Supplementary Table 1**. Immuno-fluorescent dyes and stains used for high content imaging.

| Staining panels | Dye, antibody | Wavelength  (excitation, emission) | Target Structure |
| --- | --- | --- | --- |
| 1 | Anti-γ-H2.AX antibody  (Abcam; #ab81299) | FITC (475/34, 536/40) | Damaged DNA strands |
|  | Cardiac Troponin T Monoclonal Antibody (13-11)  (Invitrogen™) | FITC (475/34, 536/40) | Sarcomere |
|  | MitoTracker Red CMXRos  (Invitrogen™) | Texas Red (560/32, 593/40) | Mitochondria |
|  | DAPI  (Invitrogen™) | DAPI (377/54, 447/60) | Nuclei |
| 2 | Anti-fibrillarin antibody  (Abcam; #ab4566) | FITC (475/34, 536/40) | Nucleoli |
|  | LysoTracker Red  (Invitrogen™) | Texas Red (560/32, 593/40) | Lysosomes |
|  | Anti-Cxc43 antibody  (Sigma Aldrich; #ZRB1179) | Cy5 (631/28, 624/40) | Gap junctions |
|  | DAPI  (Invitrogen™, #D3571) | DAPI (377/54, 447/60) | Nuclei |
| 3 | Concanavalin A, Texas Red  (Invitrogen™) | Texas Red (560/32, 593/40) | Endoplasmic reticulum |
|  | Wheat Germ agglutinin (WGA) 555  (Invitrogen™) |  | Golgi |
|  | Anti-PMP70 antibody  (Invitrogen™, #ARC2131) | FITC (475/34, 536/40) | Peroxisomes |
|  | DAPI  (Invitrogen™) | DAPI (377/54, 447/60) | Nuclei |

**Supplementary Table 2**. List of serum-free medium combinations tested.

| Basal medium | Serum-free supplement |
| --- | --- |
| Standard serum-containing medium* | Bovine serum albumin (BSA)(Huebsch et al., 2022) |
| Maturation-promoting serum-free medium* | Knockout-serum replacement (KOSR)(Zhang et al., 2021) |
| Serum-free maintenance medium(Ng et al., 2008)** | Human recombinant albumin (rHA)(Burridge et al., 2014) |
|  | Pannexin Basic |
|  | Pannexin Chemically Defined (CS) |
|  | Combination of BSA and KOSR (Feyen et al., 2020) |

*****Ncardia’s proprietary medium compositions
**Medium composition is equivalent to BPEL

**Supplementary Table 3**. hiPSC-CM line information.

| Cell line | Ncyte CM | NCRM-5 | NC196 |
| --- | --- | --- | --- |
| Somatic cell origin | Renal epithelial | Cord blood | Mononuclear blood cells (MBCs) |
| Donor gender | Female | Male | Female |
| Pathology | Healthy | Healthy | Healthy |
| Reprogramming method | Episomal | Episomal | Episomal |
| Provider | Mononuclear blood cells (MBCs) | RUCDR Infinite Biologics | Maastricht University |
| Differentiation protocol | 3D bioreactor derived | 3D bioreactor derived | 2D monolayer |
| hiPSC-CM purity* | Per batch: CM1 ≥84%  CM2 ≥95% | ≥82% | ≥79% |

*hiPSC-CM purity based on cardiac troponin T positivity assessed using flow cytometry as described above.

**Supplementary Table 4**. Electrophysiology parameters recorded after compound addition.

| Compound | Concentration (µM) | BPM | BPM - SD | FPDc | FPDc -SD | FPD | | FPD - SD | |
| --- | --- | --- | --- | --- | --- | --- | --- | --- | --- |
| Methotrexate (MTX) | 0.001 | -1.4 | 9.1 | -2.1 | 3.9 | -6.1 | | 6.3 | |
|  | 0.003 | -9.3 | 3.4 | 5.0 | 2.4 | 5.8 | | 2.5 | |
|  | 0.01 | -4.0 | 10.2 | 5.7 | 2.9 | 7.2 | | 3.0 | |
|  | 0.03 | -8.0 | 7.0 | 6.0 | 4.4 | 5.5 | | 7.0 | |
|  | 0.1 | -6.2 | 5.9 | 3.3 | 2.3 | 6.2 | | 3.3 | |
|  | 0.3 | -10.6 | 2.8 | 5.7 | 3.6 | 7.1 | | 3.5 | |
|  | 1 | -3.8 | 2.8 | -0.5 | 7.2 | 4.1 | | 4.1 | |
| Omecamtiv Mecarbil | 0.001 | -9.8 | 7.4 | 1.8 | 4.7 | 4.0 | | 6.9 | |
|  | 0.003 | -15.0 | 1.2 | 8.9 | 3.5 | 13.0 | | 4.1 | |
|  | 0.01 | -10.9 | 6.1 | 5.8 | 4.0 | 8.6 | | 6.1 | |
|  | 0.03 | 2.5 | 9.4 | 0.1 | 4.3 | -1.1 | | 6.5 | |
|  | 0.1 | -6.1 | 3.9 | 6.2 | 4.5 | 7.4 | | 5.3 | |
|  | 0.3 | -5.5 | 5.4 | 3.6 | 5.3 | 4.5 | | 6.8 | |
|  | 1 | 9.5 | 4.4 | -7.7 | 3.8 | -10.8 | | 4.0 | |
| 5-FU | 0.01 | -1.9 | 6.6 | 5.0 | 6.1 | 0.0 | | 7.6 | |
|  | 0.03 | -2.8 | 5.2 | 7.6 | 1.4 | 2.7 | | 2.1 | |
|  | 0.1 | -7.6 | 2.9 | 11.6 | 5.6 | 8.1 | | 6.6 | |
|  | 0.3 | -1.4 | 7.9 | 11.5 | 2.0 | 6.5 | | 3.6 | |
|  | 1 | -8.3 | 6.8 | 14.7 | 5.2 | 11.6 | 7.1 | |  |
|  | 3 | -5.5 | 6.2 | 9.3 | 5.3 | 5.3 | 6.7 | |  |
|  | 10 | -2.6 | 1.7 | 0.9 | 2.8 | 1.3 | 2.8 | |  |
| Cisplatin | 0.01 | -1.2 | 10.0 | 3.4 | 5.4 | 3.9 | 8.0 | |  |
|  | 0.03 | -6.9 | 1.5 | 5.6 | 3.3 | 7.3 | 3.3 | |  |
|  | 0.1 | -12.2 | 3.6 | 9.2 | 2.3 | 12.6 | 3.4 | |  |
|  | 0.3 | -4.4 | 4.3 | 5.8 | 2.7 | 6.9 | 3.8 | |  |
|  | 1 | -0.2 | 7.3 | 3.7 | 4.4 | 3.8 | 6.0 | |  |
|  | 3 | 10.8 | 4.6 | -3.0 | 0.9 | -5.4 | 1.0 | |  |
|  | 10 | 24.1 | 9.4 | -22.5 | 3.0 | -26.7 | 4.1 | |  |
| Doxorubicin | 0.01 | -7.5 | 6.8 | 2.8 | 2.0 | 4.7 | 3.6 | |  |
|  | 0.03 | 10.1 | 9.4 | -3.2 | 2.8 | -5.4 | 4.6 | |  |
|  | 0.1 | 52.1 | 12.4 | -22.2 | 5.2 | -29.7 | 6.1 | |  |
|  | 0.3 | 79.6 | 2.0 | -20.5 | 3.7 | -30.9 | 3.1 | |  |
|  | 1 | 49.1 | 16.2 | -38.1 | 5.2 | -44.2 | 6.0 | |  |
|  | 3 | 80.5 | 1.6 | -24.3 | 1.0 | -34.4 | 0.7 | |  |
|  | 10 | 15.9 | 34.4 | -31.1 | 5.8 | -30.8 | 11.8 | |  |
| Lapatinib | 0.01 | -1.6 | 5.7 | 1.1 | 3.7 | 1.6 | 5.2 | |  |
|  | 0.03 | -14.1 | 6.4 | 14.6 | 4.0 | 19.4 | 6.5 | |  |
|  | 0.1 | -10.6 | 5.4 | 12.7 | 5.4 | 16.1 | 7.3 | |  |
|  | 0.3 | -14.2 | 6.1 | 15.7 | 5.9 | 20.5 | 7.8 | |  |
|  | 1 | -14.9 | 2.2 | 13.3 | 2.4 | 18.1 | 2.7 | |  |
|  | 3 | -12.4 | 3.2 | 2.7 | 8.2 | 6.2 | 8.6 | |  |
|  | 10 | -8.4 | 7.6 | -22.1 | 3.9 | -20.8 | 5.4 | |  |
| Bupivacaine | 0.01 | -0.9 | 4.4 | -0.5 | 6.0 | -0.2 | 7.2 | |  |
|  | 0.03 | -5.5 | 5.5 | 4.8 | 3.1 | 6.5 | 4.7 | |  |
|  | 0.1 | -7.6 | 3.3 | 5.1 | 2.3 | 7.2 | 3.2 | |  |
|  | 0.3 | -6.6 | 4.9 | 9.5 | 2.7 | 11.5 | 3.7 | |  |
|  | 1 | -14.0 | 2.0 | 8.8 | 6.8 | 13.1 | 7.2 | |  |
|  | 3 | -33.6 | 2.9 | -9.4 | 2.9 | 0.2 | 4.3 | |  |
|  | 10 | -39.8 | 15.6 | 9.4 | 19.4 | 24.7 | 15.1 | |  |
| Amiodarone | 0.01 | -0.6 | 6.1 | 14.4 | 3.1 | 3.5 | 4.6 | |  |
|  | 0.03 | -7.6 | 4.1 | 15.8 | 3.5 | 6.7 | 3.3 | |  |
|  | 0.1 | -13.2 | 5.6 | 12.9 | 18.1 | 5.2 | 18.1 | |  |
|  | 0.3 | -20.9 | 10.1 | -2.2 | 7.1 | -8.1 | 10.0 | |  |
|  | 1 | -44.7 | 6.6 | -12.3 | 4.3 | -10.9 | 7.3 | |  |
|  | 3 | **ND** | **ND** | **ND** | **ND** | **ND** | **ND** | |  |
|  | 10 | **ND** | **ND** | **ND** | **ND** | **ND** | **ND** | |  |
| ASA | 0.01 | 1.7 | 4.6 | 4.1 | 3.8 | 3.6 | 5.1 | |  |
|  | 0.03 | -6.7 | 1.3 | 2.4 | 3.7 | 4.2 | 3.8 | |  |
|  | 0.1 | -0.8 | 9.6 | 3.2 | 2.1 | 3.5 | 4.4 | |  |
|  | 0.3 | -6.1 | 4.3 | 4.0 | 3.2 | 5.7 | 3.6 | |  |
|  | 1 | -10.4 | 2.6 | 9.3 | 1.8 | 12.6 | 2.5 | |  |
|  | 3 | -7.0 | 6.3 | 4.6 | 3.0 | 6.7 | 5.0 | |  |
|  | 10 | 2.3 | 11.1 | -0.2 | 7.6 | -0.6 | 11.0 | |  |
| Propofol | 0.1 | 1.4 | 4.0 | 11.2 | 2.9 | -1.9 | 3.4 | |  |
|  | 0.3 | -10.9 | 2.5 | 21.4 | 0.9 | 12.3 | 1.2 | |  |
|  | 1 | -4.2 | 5.2 | 17.2 | 7.2 | 5.8 | 7.7 | |  |
|  | 3 | -5.3 | 2.4 | 19.2 | 3.0 | 8.2 | 3.7 | |  |
|  | 10 | -5.8 | 3.4 | 17.5 | 3.0 | 6.6 | 3.7 | |  |
|  | 30 | -8.0 | 4.6 | 7.9 | 5.4 | -2.8 | 7.0 | |  |
|  | 100 | -0.9 | 6.4 | -15.2 | 1.8 | -15.5 | 2.6 | |  |
| Erlotinib | 0.01 | -5.1 | 5.9 | 5.5 | 3.3 | 7.0 | 5.2 | |  |
|  | 0.03 | -10.0 | 2.9 | 7.9 | 5.6 | 11.0 | 5.4 | |  |
|  | 0.1 | -14.7 | 5.4 | 15.9 | 1.9 | 21.2 | 3.7 | |  |
|  | 0.3 | -20.4 | 7.4 | 23.8 | 4.9 | 32.2 | 8.3 | |  |
|  | 1 | -18.8 | 4.4 | 21.7 | 4.4 | 28.9 | 5.3 | |  |
|  | 3 | -11.4 | 3.2 | 12.6 | 5.4 | 16.5 | 6.6 | |  |
|  | 10 | -6.6 | 9.7 | 9.5 | 10.2 | 12.1 | 14.1 | |  |
| Dasatinib | 0.001 | -4.8 | 6.2 | 5.8 | 3.0 | 7.2 | 3.3 | |  |
|  | 0.003 | -6.3 | 4.0 | 3.0 | 3.8 | 4.7 | 4.4 | |  |
|  | 0.01 | -5.8 | 7.9 | 2.8 | 4.0 | 4.5 | 5.9 | |  |
|  | 0.03 | -5.2 | 16.4 | 4.2 | 9.4 | 6.4 | 13.3 | |  |
|  | 0.1 | -15.3 | 5.1 | 10.6 | 3.4 | 15.3 | 5.1 | |  |
|  | 0.3 | -4.0 | 6.0 | -0.2 | 2.2 | 0.9 | 3.7 | |  |
|  | 1 | 23.6 | 18.2 | -14.6 | 6.6 | -18.7 | 9.3 | |  |
| Ponatinib | 0.001 | -7.3 | 5.0 | 5.2 | 4.3 | 7.3 | 5.8 | |  |
|  | 0.003 | -0.7 | 6.2 | 1.3 | 3.5 | 1.5 | 3.8 | |  |
|  | 0.01 | -7.9 | 7.0 | 3.5 | 2.8 | 5.7 | 4.4 | |  |
|  | 0.03 | -14.6 | 5.2 | 7.4 | 5.6 | 11.7 | 7.4 | |  |
|  | 0.1 | -21.1 | 1.9 | 6.0 | 5.4 | 12.3 | 6.1 | |  |
|  | 0.3 | -33.7 | 2.7 | 2.7 | 3.3 | 13.4 | 4.2 | |  |
|  | 1 | -43.4 | 5.3 | -15.5 | 1.6 | -2.9 | 2.7 | |  |
| Chlorpromazine | 0.001 | -5.4 | 6.9 | 14.8 | 2.7 | 6.0 | 4.3 | |  |
|  | 0.003 | -13.2 | 6.4 | 18.0 | 3.5 | 11.6 | 5.4 | |  |
|  | 0.01 | -17.7 | 3.8 | 22.9 | 3.9 | 18.1 | 5.0 | |  |
|  | 0.03 | -13.3 | 2.5 | 23.2 | 0.8 | 17.0 | 0.8 | |  |
|  | 0.1 | -14.0 | 3.1 | 20.0 | 2.7 | 13.9 | 3.8 | |  |
|  | 0.3 | -15.0 | 5.5 | 22.5 | 7.0 | 16.9 | 9.1 | |  |
|  | 1 | -9.3 | 9.6 | -2.0 | 10.8 | 0.6 | 13.0 | |  |
| Digoxin | 1.00E-05 | 5.3 | 16.8 | 13.3 | 7.0 | -0.8 | 10.7 | |  |
|  | 3.00E-05 | -8.4 | 1.9 | 18.7 | 1.7 | 7.9 | 2.1 | |  |
|  | 1.00E-04 | -3.8 | 8.4 | 18.4 | 5.6 | 6.4 | 6.7 | |  |
|  | 3.00E-04 | -8.1 | 2.4 | 17.7 | 2.7 | 6.8 | 3.0 | |  |
|  | 0.001 | 4.0 | 9.3 | 9.9 | 3.6 | -4.4 | 5.6 | |  |
|  | 0.003 | 15.3 | 6.7 | -9.3 | 2.7 | -26.1 | 3.3 | |  |
|  | 0.01 | 23.3 | 11.1 | -65.7 | 3.0 | -69.7 | 3.7 | |  |
| Dofetilide | 1.00E-04 | 1.5 | 5.1 | -1.9 | 4.5 | -2.3 | 5.6 | |  |
|  | 3.00E-04 | -3.1 | 3.5 | 3.8 | 2.7 | 4.8 | 3.9 | |  |
|  | 0.001 | -2.8 | 2.6 | 8.0 | 2.5 | 9.0 | 3.3 | |  |
|  | 0.003 | -9.3 | 6.1 | 18.6 | 7.0 | 22.2 | 9.3 | |  |
|  | 0.01 | -11.5 | 17.0 | 55.3 | 17.9 | 63.2 | 25.1 | |  |
|  | 0.03 | -42.2 | 4.2 | -13.8 | 9.6 | -1.4 | 11.9 | |  |
|  | 0.1 | -65.4 | 0.0 | 17.6 | 0.0 | 59.9 | 0.0 | |  |
| Empagliflozin | 0.001 | -3.0 | 8.4 | 2.7 | 6.2 | 3.8 | 8.7 | |  |
|  | 0.003 | 2.0 | 7.0 | 1.0 | 3.9 | 0.6 | 5.4 | |  |
|  | 0.01 | -3.8 | 11.0 | 8.3 | 6.6 | 10.0 | 9.9 | |  |
|  | 0.03 | -1.2 | 7.5 | 0.8 | 2.5 | 1.2 | 4.3 | |  |
|  | 0.1 | -2.9 | 4.2 | 3.0 | 2.5 | 3.9 | 3.4 | |  |
|  | 0.3 | -6.0 | 2.9 | 6.4 | 3.4 | 8.2 | 3.4 | |  |
|  | 1 | -6.8 | 9.0 | 6.2 | 6.7 | 8.6 | 8.4 | |  |

*****BPM: beat per minute, FPD: field potential duration, FPDc: corrected field potential duration, SD: standard deviation. Values shown as percentage changes (Δ%) relative to baseline.

**Supplementary Table 5**. List of imaging parameters extracted from the MetaXpress image analysis tool after filtering for near zero variance and removing redundant parameters.

| Cytoplasm_Total_Area_Sum | Nuclei_Shape_Factor_Sum.1 |
| --- | --- |
| Cytoplasm_Minimum_Intensity_Sum | Nuclei_Fiber_Length_Average.1 |
| Cytoplasm_Maximum_Intensity_Sum | Nuclei_Fiber_Length_Sum.1 |
| Cytoplasm_Width_Sum | Nuclei_Fiber_Breadth_Average.1 |
| Cytoplasm_Height_Sum | Nuclei_Fiber_Breadth_Sum.1 |
| Cytoplasm_Average_Intensity_Sum | Nuclei_Breadth_Average.1 |
| Cytoplasm_Intensity_Std._Dev._Sum | Nuclei_Breadth_Sum.1 |
| Cytoplasm_Length_Sum | Lysosomes_per_Cell |
| Nuclei_Area_Average | Lysosomes_Minimum_Intensity_Average |
| Nuclei_Width_Average | Lysosomes_Length_Average |
| Nuclei_Height_Average | Lysosomes_Intensity_Std._Dev._Average |
| Nuclei_Integrated_Intensity_Average | Nucleoli_Intensity_Std._Dev._Average |
| Nuclei_Intensity_Std._Dev._Average | Nucleoli_Minimum_Intensity_Average |
| Nuclei_Minimum_Intensity_Average | Nucleoli_Maximum_Intensity_Average |
| Nuclei_Length_Average | Nucleoli_Length_Average |
| Nuclei_Total_Area_Sum | Nucleoli_count |
| Nuclei_Width_Sum | Lysosomes_Intensity_Std._Dev._Sum |
| Nuclei_Height_Sum | Nucleoli_Intensity_Std._Dev._Sum |
| Nuclei_Integrated_Intensity_Sum | Cxc43_Integrated_Intensity_Average |
| Nuclei_Average_Intensity_Sum | Cxc43_Intensity_Std._Dev._Average |
| Nuclei_Intensity_Std._Dev._Sum | Cxc43_Minimum_Intensity_Average |
| Nuclei_Minimum_Intensity_Sum | Cxc43_Maximum_Intensity_Average |
| Nuclei_Maximum_Intensity_Sum | Cxc43_Count_Sum |
| Nuclei_Length_Sum | Nucleoli_Maximum_Intensity_Sum |
| Cytoplasm_Integrated_Intensity_Sum | Lysosomes_Integrated_Intensity_Average |
| Cytoplasm_Perimeter_Sum | Lysosomes_Integrated_Intensity_Sum |
| Cytoplasm_Shape_Factor_Sum | Lysosomes_Perimeter_Average |
| Cytoplasm_Fiber_Breadth_Sum | Lysosomes_Shape_Factor_Average |
| Cytoplasm_Breadth_Sum | Lysosomes_Fiber_Length_Average |
| Nuclei_Shape_Factor_Average | Lysosomes_Fiber_Breadth_Average |
| Nuclei_Shape_Factor_Sum | Lysosomes_Breadth_Sum |
| Nuclei_Fiber_Length_Average | Nucleoli_Integrated_Intensity_Sum |
| Nuclei_Fiber_Length_Sum | Nucleoli_Perimeter_Average |
| Nuclei_Fiber_Breadth_Average | Nucleoli_Shape_Factor_Average |
| Nuclei_Fiber_Breadth_Sum | Nucleoli_Fiber_Length_Average |
| Nuclei_Breadth_Average | Nucleoli_Fiber_Breadth_Average |
| Nuclei_Breadth_Sum | Nucleoli_Breadth_Sum |
| Mitochondria_Width_Average | Cxc43_Perimeter_Average |
| Mitochondria_Minimum_Intensity_Average | Cxc43_Shape_Factor_Average |
| Mitochondria_Length_Average | Cxc43_Shape_Factor_Sum |
| Mitochondria_Intensity_Std._Dev._Average | Cxc43_Fiber_Length_Average |
| DNA_damage_Area_Average | Cxc43_Fiber_Breadth_Average |
| DNA_damage_Width_Average | Cxc43_Breadth_Average |
| DNA_damage_Height_Average | Cytoplasm_Area_Sum_cell.2 |
| DNA_damage_Intensity_Std._Dev._Average | Cytoplasm_Minimum_Intensity_Sum.2 |
| DNA_damage_Minimum_Intensity_Average | Cytoplasm_Maximum_Intensity_Sum.2 |
| DNA_damage_Length_Average | Cytoplasm_Width_Sum.2 |
| DNA_damage_count | Cytoplasm_Height_Sum.2 |
| DNA_damage_Intensity_Std._Dev._Sum | Cytoplasm_Average_Intensity_Sum.2 |
| Mitochondria_Breadth_Sum | Cytoplasm_Intensity_Std._Dev._Sum.2 |
| DNA_damage_Perimeter_Average | Cytoplasm_Length_Sum.2 |
| DNA_damage_Shape_Factor_Average | Nuclei_Area_Average.2 |
| DNA_damage_Shape_Factor_Sum | Nuclei_Width_Average.2 |
| DNA_damage_Fiber_Length_Average | Nuclei_Height_Average.2 |
| DNA_damage_Breadth_Average | Nuclei_Integrated_Intensity_Average.2 |
| Sarcomere_Total_Area_Average | Nuclei_Intensity_Std._Dev._Average.2 |
| Sarcomere_Area_Sum | Nuclei_Minimum_Intensity_Average.2 |
| Sarcomere_Width_Sum | Nuclei_Maximum_Intensity_Average.2 |
| Sarcomere_Height_Average | Nuclei_Total_Area_Sum.2 |
| Sarcomere_Height_Sum | Nuclei_Width_Sum.2 |
| Sarcomere_Integrated_Intensity_Average | Nuclei_Height_Sum.2 |
| Sarcomere_Integrated_Intensity_Sum | Nuclei_Integrated_Intensity_Sum.2 |
| Sarcomere_Average_Intensity_Average | Nuclei_Average_Intensity_Sum.2 |
| Sarcomere_Intensity_Std._Dev._Average | Nuclei_Intensity_Std._Dev._Sum.2 |
| Sarcomere_Minimum_Intensity_Average | Nuclei_Minimum_Intensity_Sum.2 |
| Sarcomere_Minimum_Intensity_Sum | Nuclei_Maximum_Intensity_Sum.2 |
| Sarcomere_Perimeter_Sum | Nuclei_Length_Sum.2 |
| Sarcomere_Shape_Factor_Average | ER_._Golgi_Width_Average |
| Sarcomere_Shape_Factor_Sum | ER_._Golgi_Height_Average |
| Sarcomere_Fiber_Length_Average | ER_._Golgi_Minimum_Intensity_Average |
| Sarcomere_Fiber_Breadth_Average | ER_._Golgi_Length_Average |
| Sarcomere_Fiber_Breadth_Sum | ER_._Golgi_Intensity_Std._Dev._Average |
| Sarcomere_Length_Average | Peroxisomes_Total_Area_Average |
| Sarcomere_Features_Count_Sum | Peroxisomes_Width_Average |
| Cytoplasm_Total_Area_Sum.1 | Peroxisomes_Height_Average |
| Cytoplasm_Minimum_Intensity_Sum.1 | Peroxisomes_Intensity_Std._Dev._Average |
| Cytoplasm_Width_Sum.1 | Peroxisomes_Minimum_Intensity_Average |
| Cytoplasm_Height_Sum.1 | Peroxisomes_Length_Average |
| Cytoplasm_Average_Intensity_Sum.1 | ER_._Golgi_Minimum_Intensity_Sum |
| Cytoplasm_Intensity_Std._Dev._Sum.1 | Peroxisomes_Minimum_Intensity_Sum |
| Cytoplasm_Length_Sum.1 | Cytoplasm_Integrated_Intensity_Sum.2 |
| Nuclei_Total_Area_Average.1 | Cytoplasm_Perimeter_Sum.2 |
| Nuclei_Width_Average.1 | Cytoplasm_Shape_Factor_Sum.2 |
| Nuclei_Height_Average.1 | Cytoplasm_Fiber_Breadth_Sum.2 |
| Nuclei_Integrated_Intensity_Average.1 | Cytoplasm_Breadth_Sum.2 |
| Nuclei_Intensity_Std._Dev._Average.1 | Nuclei_Shape_Factor_Average.2 |
| Nuclei_Minimum_Intensity_Average.1 | Nuclei_Shape_Factor_Sum.2 |
| Nuclei_Length_Average.1 | Nuclei_Fiber_Length_Average.2 |
| Nuclei_Total_Area_Sum.1 | Nuclei_Fiber_Length_Sum.2 |
| Nuclei_Width_Sum.1 | Nuclei_Fiber_Breadth_Average.2 |
| Nuclei_Height_Sum.1 | Nuclei_Fiber_Breadth_Sum.2 |
| Nuclei_Integrated_Intensity_Sum.1 | Nuclei_Breadth_Average.2 |
| Nuclei_Intensity_Std._Dev._Sum.1 | Nuclei_Breadth_Sum.2 |
| Nuclei_Minimum_Intensity_Sum.1 | ER_._Golgi_Integrated_Intensity_Average |
| Nuclei_Maximum_Intensity_Sum.1 | ER_._Golgi_Integrated_Intensity_Sum |
| Nuclei_Length_Sum.1 | ER_._Golgi_Shape_Factor_Average |
| Cytoplasm_Integrated_Intensity_Sum.1 | ER_._Golgi_Shape_Factor_Sum |
| Cytoplasm_Shape_Factor_Sum.1 | ER_._Golgi_Fiber_Length_Sum |
| Cytoplasm_Fiber_Length_Sum.1 | ER_._Golgi_Fiber_Breadth_Average |
| Cytoplasm_Fiber_Breadth_Sum.1 | ER_._Golgi_Breadth_Average |
| Cytoplasm_Breadth_Sum.1 | Peroxisomes_Shape_Factor_Average |
| Nuclei_Perimeter_Average.1 | Peroxisomes_Shape_Factor_Sum |
| Nuclei_Shape_Factor_Average.1 |  |

**Supplementary Table 6**. List of available default features in the MetaXpress software using the Custom Module editor.

| Measurement Name | Average Column | Sum Column | Description |
| --- | --- | --- | --- |
| Total Area | Total Area_Average | Total Area_Sum | Area of the entire object |
| Hole Area | Hole Area_Average | Hole Area_Sum | Area of the hole in the object of feature |
| Area | Area_Average | Area_Sum | Area of the object (excluding holes) |
| Relative Hole Area | Relative Hole Area_Average | Relative Hole Area_Sum | Ratio of the hole area to the total area |
| Standard Area Count | Standard Area Count_Average | Standard Area Count_Sum | Number of times larger than the object to measure |
| Width | Width_Average | Width_Sum | Horizontal dimension of the object |
| Height | Height_Average | Height_Sum | Vertical dimension of the object |
| Centroid X | Centroid X_Average | Centroid X_Sum | X coordinates of the centroid of the object |
| Centroid Y | Centroid Y_Average | Centroid Y_Sum | Y coordinates of the centroid of the object |
| Intensity Center X | Intensity Center X_Average | Intensity Center X_Sum | X coordinates of the intensity-weighted centroid |
| Intensity Center Y | Intensity Center Y_Average | Intensity Center Y_Sum | Y coordinates of the intensity-weighted centroid |
| Integrated Intensity | Integrated Intensity_Average | Integrated Intensity_Sum | Sum of all the intensity value for the pixels in the object |
| Average Intensity | Average Intensity_Average | Average Intensity_Sum | Average of all the intensity value for the pixels in the object |
| Intensity Std. Dev. | Intensity Std. Dev._Average | Intensity Std. Dev._Sum | SD around the mean of the average intensity in the object |
| Minimum Intensity | Minimum Intensity_Average | Minimum Intensity_Sum | Minimum pixel intensity value in the object |
| Maximum Intensity | Maximum Intensity_Average | Maximum Intensity_Sum | Maximum pixel intensity value in the object |
| Perimeter | Perimeter_Average | Perimeter_Sum | Distance around the edge of the object |
| Shape Factor | Shape Factor_Average | Shape Factor_Sum | Value from 0 to 1, closest to a circle (1 is a perfect circle) |
| Fiber Length | Fiber Length_Average | Fiber Length_Sum | Length, if the object is a fiber |
| Fiber Breadth | Fiber Breadth_Average | Fiber Breadth_Sum | Breadth, if the object is a fiber |
| Length | Length_Average | Length_Sum | Span of the longest chord through the object |
| Orientation | Orientation_Average | Orientation_Sum | Angle between the longest chord and the horizontal axis |
| Breadth | Breadth_Average | Breadth_Sum | Caliper width of the object |
| Ell. Form Factor | Ell. Form Factor_Average | Ell. Form Factor_Sum | Ratio of the breadth to the length of the object |
| Pixel Centroid X | Pixel Centroid X_Average | Pixel Centroid X_Sum | X coordinates of the pixel centroid of the object |
| Pixel Centroid Y | Pixel Centroid Y_Average | Pixel Centroid Y_Sum | Y coordinates of the pixel centroid of the object |
| Line Length | Line Length_Average | Line Length_Sum | Length of the object |
| Features Count |  | Features Count_Sum | Total number of objects |

**Supplementary Table 7**. GO term analysis of additional compounds, with corresponding gene IDs.

| Compound | Categ. | ID | Term | Gene ID (ensembl) |
| --- | --- | --- | --- | --- |
| Bupivacain | BP | GO:0045010 | actin nucleation | ENSG00000163380, ENSG00000170807, ENSG00000196405 |
|  | BP | GO:0007051 | spindle organization | ENSG00000138160, ENSG00000066279 |
|  | BP | GO:0097150 | neuronal stem cell population maintenance | ENSG00000066279, ENSG00000144554 |
|  | BP | GO:0048589 | developmental growth | ENSG00000066279, ENSG00000112658 |
|  | CC | GO:0031430 | M band | ENSG00000074800, ENSG00000163380, ENSG00000170807 |
|  | CC | GO:0030017 | sarcomere | ENSG00000143632, ENSG00000174429, ENSG00000170807 |
|  | CC | GO:0005813 | centrosome | ENSG00000137807, ENSG00000066279, ENSG00000166803, ENSG00000159399, ENSG00000072571, ENSG00000148019, ENSG00000143476 |
|  | CC | GO:0005865 | striated muscle thin filament | ENSG00000143632, ENSG00000163380, ENSG00000170807 |
|  | MF | GO:0005324 | long-chain fatty acid transporter activity | ENSG00000117394, ENSG00000143554 |
|  | MF | GO:0005515 | protein binding | ENSG00000074800, ENSG00000129521, ENSG00000197299, ENSG00000197893, ENSG00000213347, ENSG00000006634, ENSG00000111674, ENSG00000177606, ENSG00000137267, ENSG00000196405, ENSG00000114023, ENSG00000143632, ENSG00000170525, ENSG00000151729, ENSG00000124839, ENSG00000141295, ENSG00000166592, ENSG00000136492, ENSG00000126458, ENSG00000127824, ENSG00000180448, ENSG00000081181, ENSG00000143476, ENSG00000167767, ENSG00000156970, ENSG00000159200, ENSG00000111665, ENSG00000113368, ENSG00000242114, ENSG00000152256, ENSG00000176208, ENSG00000198929, ENSG00000138160, ENSG00000186352, ENSG00000088325, ENSG00000163975, ENSG00000166803, ENSG00000141526, ENSG00000118729, ENSG00000092853, ENSG00000144554, ENSG00000129534, ENSG00000137807, ENSG00000154188, ENSG00000176014, ENSG00000171320, ENSG00000159399, ENSG00000134333, ENSG00000189057, ENSG00000174429, ENSG00000163380, ENSG00000113070, ENSG00000117394, ENSG00000177181, ENSG00000162772, ENSG00000176171, ENSG00000106462, ENSG00000123485, ENSG00000102144, ENSG00000109805, ENSG00000112658, ENSG00000134222, ENSG00000072571, ENSG00000120937, ENSG00000137812, ENSG00000159348, ENSG00000196968, ENSG00000169855, ENSG00000142871, ENSG00000167552, ENSG00000171848, ENSG00000123219, ENSG00000185567, ENSG00000148773 |
| Chlorpromazine | BP | GO:0051301 | cell division | ENSG00000138182, ENSG00000109805, ENSG00000066279, ENSG00000088325, ENSG00000111665 |
|  | BP | GO:0007049 | cell cycle | ENSG00000138182, ENSG00000111665, ENSG00000148773, ENSG00000123485 |
|  | CC | GO:0005829 | cytosol | ENSG00000143632, ENSG00000129991, ENSG00000138182, ENSG00000109805, ENSG00000171848, ENSG00000088325, ENSG00000111674, ENSG00000111665, ENSG00000185567 |
|  | CC | GO:0005634 | nucleus | ENSG00000138182, ENSG00000109805, ENSG00000171848, ENSG00000066279, ENSG00000088325, ENSG00000185567, ENSG00000148773, ENSG00000120937, ENSG00000123485 |
| Cisplatin | BP | GO:0086091 | regulation of heart rate by cardiac conduction | ENSG00000130037, ENSG00000053918, ENSG00000006283, ENSG00000183230 |
|  | BP | GO:0007155 | cell adhesion | ENSG00000162692, ENSG00000169862, ENSG00000204963, ENSG00000183230, ENSG00000154229, ENSG00000049130, ENSG00000116117 |
|  | BP | GO:0016477 | cell migration | ENSG00000183098, ENSG00000183230, ENSG00000167779, ENSG00000137642 |
|  | CC | GO:0009986 | cell surface | ENSG00000162692, ENSG00000130037, ENSG00000053918, ENSG00000183098, ENSG00000134243, ENSG00000178726, ENSG00000137642 |
|  | CC | GO:0005794 | Golgi apparatus | ENSG00000110328, ENSG00000135604, ENSG00000162692, ENSG00000130037, ENSG00000130513, ENSG00000185274, ENSG00000180263, ENSG00000134243, ENSG00000185532, ENSG00000112984, ENSG00000167779, ENSG00000137642 |
|  | CC | GO:0045202 | synapse | ENSG00000182836, ENSG00000006283, ENSG00000183098, ENSG00000157680, ENSG00000165617, ENSG00000146555 |
|  | CC | GO:0005886 | plasma membrane | ENSG00000135604, ENSG00000130037, ENSG00000053918, ENSG00000107738, ENSG00000134243, ENSG00000178726, ENSG00000170310, ENSG00000154229, ENSG00000206561, ENSG00000150672, ENSG00000137642, ENSG00000162692, ENSG00000080031, ENSG00000169862, ENSG00000027075, ENSG00000183098, ENSG00000157680, ENSG00000204963, ENSG00000006283, ENSG00000185532, ENSG00000049130, ENSG00000146555 |
|  | MF | GO:0005515 | protein binding | ENSG00000130037, ENSG00000135604, ENSG00000182836, ENSG00000130513, ENSG00000107738, ENSG00000170310, ENSG00000154229, ENSG00000112984, ENSG00000100918, ENSG00000137642, ENSG00000080031, ENSG00000166851, ENSG00000169862, ENSG00000134690, ENSG00000027075, ENSG00000183098, ENSG00000183230, ENSG00000185532, ENSG00000130429, ENSG00000184205, ENSG00000186628, ENSG00000104313, ENSG00000053918, ENSG00000156475, ENSG00000064692, ENSG00000134243, ENSG00000178726, ENSG00000112699, ENSG00000116117, ENSG00000120937, ENSG00000159147, ENSG00000153820, ENSG00000150672, ENSG00000206561, ENSG00000186073, ENSG00000183580, ENSG00000157680, ENSG00000165617, ENSG00000049130, ENSG00000101144, ENSG00000183049, ENSG00000167779, ENSG00000143320 |
| Dasatinib | BP | GO:0051726 | regulation of cell cycle | ENSG00000071246, ENSG00000075624, ENSG00000138182, ENSG00000198873, ENSG00000166803, ENSG00000099860, ENSG00000108984 |
|  | BP | GO:0010811 | positive regulation of cell-substrate adhesion | ENSG00000144810, ENSG00000091986 |
|  | MF | GO:0005515 | protein binding | ENSG00000137807, ENSG00000149591, ENSG00000176014, ENSG00000111674, ENSG00000099860, ENSG00000150687, ENSG00000111057, ENSG00000137801, ENSG00000171345, ENSG00000004799, ENSG00000196405, ENSG00000170017, ENSG00000136732, ENSG00000143632, ENSG00000122952, ENSG00000141295, ENSG00000087303, ENSG00000136492, ENSG00000185950, ENSG00000091039, ENSG00000186205, ENSG00000108984, ENSG00000123485, ENSG00000106366, ENSG00000071246, ENSG00000129991, ENSG00000075624, ENSG00000139618, ENSG00000113448, ENSG00000110436, ENSG00000198873, ENSG00000111665, ENSG00000120937, ENSG00000170421, ENSG00000144810, ENSG00000118523, ENSG00000137331, ENSG00000138182, ENSG00000138160, ENSG00000171848, ENSG00000147883, ENSG00000166803, ENSG00000185567, ENSG00000181649 |
|  | MF | GO:0001968 | fibronectin binding | ENSG00000118523, ENSG00000137801, ENSG00000091986 |
| Digoxin | BP | GO:0045444 | fat cell differentiation | ENSG00000198873, ENSG00000119508, ENSG00000188735, ENSG00000123358, ENSG00000171488 |
|  | BP | GO:0001666 | response to hypoxia | ENSG00000148926, ENSG00000117394, ENSG00000112658, ENSG00000159399, ENSG00000176171 |
|  | BP | GO:0019934 | cGMP-mediated signaling | ENSG00000072952, ENSG00000120937, ENSG00000152402 |
|  | BP | GO:0007165 | signal transduction | ENSG00000148926, ENSG00000197471, ENSG00000164761, ENSG00000130513, ENSG00000120217, ENSG00000119508, ENSG00000172575, ENSG00000114646, ENSG00000104419, ENSG00000123358, ENSG00000180448, ENSG00000152402 |
|  | BP | GO:0045671 | negative regulation of osteoclast differentiation | ENSG00000104332, ENSG00000152154, ENSG00000164761 |
|  | BP | GO:0044344 | cellular response to fibroblast growth factor stimulus | ENSG00000104332, ENSG00000026508, ENSG00000123358 |
|  | BP | GO:0001570 | vasculogenesis | ENSG00000148926, ENSG00000157483, ENSG00000171223 |
|  | BP | GO:0046330 | positive regulation of JNK cascade | ENSG00000172575, ENSG00000135299, ENSG00000172985 |
|  | BP | GO:0007264 | small GTPase mediated signal transduction | ENSG00000138193, ENSG00000166592, ENSG00000172575 |
|  | CC | GO:0005737 | cytoplasm | ENSG00000111913, ENSG00000130513, ENSG00000174611, ENSG00000164070, ENSG00000159433, ENSG00000171488, ENSG00000152402, ENSG00000148926, ENSG00000188322, ENSG00000168672, ENSG00000125148, ENSG00000162458, ENSG00000176171, ENSG00000081181, ENSG00000107796, ENSG00000072952, ENSG00000198873, ENSG00000112658, ENSG00000135299, ENSG00000104419, ENSG00000120937, ENSG00000134363, ENSG00000134369, ENSG00000258947, ENSG00000157483, ENSG00000091622, ENSG00000163833, ENSG00000123358, ENSG00000164649, ENSG00000185745 |
|  | CC | GO:0005856 | cytoskeleton | ENSG00000111913, ENSG00000109107, ENSG00000163431, ENSG00000146021, ENSG00000157483, ENSG00000174611 |
|  | CC | GO:0005789 | endoplasmic reticulum membrane | ENSG00000152154, ENSG00000074416, ENSG00000072952, ENSG00000154153, ENSG00000144959, ENSG00000172575, ENSG00000114646, ENSG00000109321, ENSG00000171488 |
|  | CC | GO:0030018 | Z disc | ENSG00000197361, ENSG00000117394, ENSG00000174611 |
|  | MF | GO:0005324 | long-chain fatty acid transporter activity | ENSG00000117394, ENSG00000113396 |
|  | MF | GO:0005515 | protein binding | ENSG00000111913, ENSG00000198780, ENSG00000130513, ENSG00000146021, ENSG00000111674, ENSG00000171223, ENSG00000138193, ENSG00000166592, ENSG00000185112, ENSG00000180448, ENSG00000081181, ENSG00000104332, ENSG00000074416, ENSG00000197361, ENSG00000100170, ENSG00000072952, ENSG00000109107, ENSG00000154153, ENSG00000104419, ENSG00000134363, ENSG00000152779, ENSG00000152256, ENSG00000188735, ENSG00000123358, ENSG00000185745, ENSG00000197471, ENSG00000104765, ENSG00000026508, ENSG00000159399, ENSG00000114646, ENSG00000171488, ENSG00000113594, ENSG00000152402, ENSG00000163661, ENSG00000174429, ENSG00000144824, ENSG00000117394, ENSG00000120217, ENSG00000168672, ENSG00000119508, ENSG00000125148, ENSG00000162458, ENSG00000105251, ENSG00000176171, ENSG00000198873, ENSG00000112658, ENSG00000120937, ENSG00000130066, ENSG00000172985, ENSG00000166343, ENSG00000164761, ENSG00000101605, ENSG00000258947, ENSG00000157483, ENSG00000091622, ENSG00000109321, ENSG00000164649 |
| Lapatinib | BP | GO:0006695 | cholesterol biosynthetic process | ENSG00000052802, ENSG00000067064, ENSG00000072310, ENSG00000147383, ENSG00000112972, ENSG00000132196, ENSG00000172893, ENSG00000131473, ENSG00000001630, ENSG00000113161, ENSG00000116133, ENSG00000186480, ENSG00000160285, ENSG00000149809, ENSG00000167508, ENSG00000079459, ENSG00000147155, ENSG00000160752 |
|  | BP | GO:0008610 | lipid biosynthetic process | ENSG00000052802, ENSG00000151726, ENSG00000072310, ENSG00000079459, ENSG00000109929, ENSG00000131473, ENSG00000131069 |
|  | CC | GO:0005783 | endoplasmic reticulum | ENSG00000113739, ENSG00000072682, ENSG00000090530, ENSG00000132196, ENSG00000104549, ENSG00000197930, ENSG00000137801, ENSG00000172893, ENSG00000137642, ENSG00000186480, ENSG00000151726, ENSG00000160097, ENSG00000091039, ENSG00000147155, ENSG00000176171, ENSG00000143554, ENSG00000102230, ENSG00000052802, ENSG00000071246, ENSG00000072310, ENSG00000133935, ENSG00000147383, ENSG00000134324, ENSG00000001630, ENSG00000113161, ENSG00000116133, ENSG00000099194, ENSG00000149809, ENSG00000109084, ENSG00000079459, ENSG00000100379, ENSG00000122884 |
|  | CC | GO:0005789 | endoplasmic reticulum membrane | ENSG00000172296, ENSG00000162407, ENSG00000134716, ENSG00000132196, ENSG00000104549, ENSG00000114646, ENSG00000197930, ENSG00000172893, ENSG00000137642, ENSG00000186480, ENSG00000151726, ENSG00000091039, ENSG00000147155, ENSG00000102230, ENSG00000052802, ENSG00000149485, ENSG00000072310, ENSG00000133935, ENSG00000147383, ENSG00000109929, ENSG00000134324, ENSG00000001630, ENSG00000113161, ENSG00000116133, ENSG00000099194, ENSG00000160285, ENSG00000149809, ENSG00000079459, ENSG00000221968 |
|  | CC | GO:0016529 | sarcoplasmic reticulum | ENSG00000213366, ENSG00000186628, ENSG00000090530, ENSG00000159399, ENSG00000118729, ENSG00000137801, ENSG00000054654 |
|  | CC | GO:0030018 | Z disc | ENSG00000100628, ENSG00000154553, ENSG00000117394, ENSG00000159176, ENSG00000118729, ENSG00000054654, ENSG00000170421, ENSG00000197616, ENSG00000196923 |
|  | CC | GO:0001725 | stress fiber | ENSG00000143632, ENSG00000154553, ENSG00000064042, ENSG00000065534, ENSG00000107796, ENSG00000197616, ENSG00000196923 |
|  | CC | GO:0005737 | cytoplasm | ENSG00000111913, ENSG00000074800, ENSG00000129521, ENSG00000064042, ENSG00000049246, ENSG00000134716, ENSG00000242265, ENSG00000113578, ENSG00000065534, ENSG00000137267, ENSG00000196405, ENSG00000197616, ENSG00000139289, ENSG00000166510, ENSG00000119408, ENSG00000120437, ENSG00000158560, ENSG00000159176, ENSG00000127824, ENSG00000100784, ENSG00000184227, ENSG00000081181, ENSG00000162433, ENSG00000102230, ENSG00000132821, ENSG00000125170, ENSG00000072310, ENSG00000137834, ENSG00000134324, ENSG00000170421, ENSG00000171314, ENSG00000169710, ENSG00000163492, ENSG00000118729, ENSG00000100379, ENSG00000054654, ENSG00000277586, ENSG00000107562, ENSG00000148677, ENSG00000140750, ENSG00000103485, ENSG00000213366, ENSG00000161958, ENSG00000149591, ENSG00000176014, ENSG00000177508, ENSG00000133169, ENSG00000131069, ENSG00000134333, ENSG00000175206, ENSG00000187678, ENSG00000147697, ENSG00000176171, ENSG00000107796, ENSG00000160752, ENSG00000132688, ENSG00000071246, ENSG00000067064, ENSG00000146072, ENSG00000173193, ENSG00000198873, ENSG00000114315, ENSG00000112972, ENSG00000261150, ENSG00000139318, ENSG00000120937, ENSG00000114796, ENSG00000131473, ENSG00000116016, ENSG00000116133, ENSG00000167553, ENSG00000157680, ENSG00000258947, ENSG00000157483, ENSG00000091622, ENSG00000119917, ENSG00000185567, ENSG00000181649 |
|  | MF | GO:0016491 | oxidoreductase activity | ENSG00000052802, ENSG00000149485, ENSG00000092621, ENSG00000132196, ENSG00000197930, ENSG00000109929, ENSG00000001630, ENSG00000171724, ENSG00000169710, ENSG00000099194, ENSG00000120658, ENSG00000145990, ENSG00000221968 |

**Supplementary Table 8**. Associated side-effect profiles of compound library.

| Drug | Drug class | FDA label warning - Cardiovascular side effects | Source |
| --- | --- | --- | --- |
| Doxorubicin | Anthracycline (chemotherapeutic) | Cardiomyopathy, heart failure, arrhythmias, myocarditis, pericarditis, and pericardial effusion. | <https://www.accessdata.fda.gov/drugsatfda_docs/label/>  2012/062921s022lbl.pdf |
| Cisplatin | Alkylating agent (chemotherapeutic) | Hypomagnesemia, hypocalcemia, and hypokalemia, which may contribute to cardiac arrhythmias. Vascular events | <https://www.accessdata.fda.gov/drugsatfda_docs/label/>  2022/018057s092lbl.pdf |
| Ponatinib | Tyrosine kinase inhibitor (chemotherapeutic) | Arterial thrombosis, myocardial infarction, heart failure, hypertension, and venous thromboembolism. Also arrhythmia | <https://www.accessdata.fda.gov/drugsatfda_docs/label/>  2024/203469s037lbl.pdf |
| Dasatinib | Tyrosine kinase inhibitor (chemotherapeutic) | Fluid retention, pericardial effusion, congestive heart failure, QT prolongation, and pulmonary arterial hypertension. | <https://www.accessdata.fda.gov/drugsatfda_docs/label/>  2024/203469s037lbl.pdf |
| Lapatinib | Tyrosine kinase inhibitor (chemotherapeutic) | Decreased LVEF and potential heart failure but also arrhythmia | <https://www.accessdata.fda.gov/drugsatfda_docs/label/>  2022/022059s031lbl.pdf |
| 5-Fluoro-uracil | Antimetabolite (chemotherapeutic) | Myocardial ischemia, myocardial infarction, angina, and arrhythmias. | <https://www.accessdata.fda.gov/drugsatfda_docs/label/>  2024/040278s027lbl.pdf |
| Methotrexate | Antimetabolite (chemotherapeutic) | Pericarditis, pericardial effusion, and hypotension. | <https://www.accessdata.fda.gov/drugsatfda_docs/label/>  2020/040054s015,s016,s017.pdf |
| Omecamtiv Mecarbil | Cardiac myosin activator | No label available | |
| Propofol | General anesthetic | Hypotension, bradycardia, and arrhythmias. | <https://www.accessdata.fda.gov/drugsatfda_docs/label/>  2022/019627s069lbl.pdf |
| Bupivacaine | Local anesthetic | Arrhythmias, hypotension, and cardiovascular collapse | <https://www.accessdata.fda.gov/drugsatfda_docs/label/>  2012/018692s015lbl.pdf |
| Amiodarone | Multiclass  antiarrhythmic | Bradycardia, heart block, QT prolongation, torsades de pointes, and hypotension. | <https://www.accessdata.fda.gov/drugsatfda_docs/label/>  2018/018972s054lbl.pdf |
| Dofetilide | Class III antiarrhythmic | QT prolongation and torsades de pointes. | <https://www.accessdata.fda.gov/drugsatfda_docs/label/>  2013/020931s007lbl.pdf |
| Digoxin | Class V antiarrhythmic | Arrhythmias, heart block, and bradycardia | <https://www.accessdata.fda.gov/drugsatfda_docs/label/>  2016/020405s013lbl.pdf |
| Chlorpromazine | Phenothiazine antipsychotics | QT prolongation, orthostatic hypotension, and tachycardia. | <https://www.accessdata.fda.gov/drugsatfda_docs/label/>  2015/203479s007s008lbl.pdf |
| Erlotinib | Tyrosine kinase inhibitor (chemotherapeutic) | Myocardial infarction and ischemia are rare but noted. | <https://www.accessdata.fda.gov/drugsatfda_docs/label/>  2010/021743s14s16lbl.pdf |
| ASA | Platelet aggregation inhibitor | Risk of bleeding may lead to hemorrhagic stroke; otherwise, it is cardioprotective at low doses. | <https://www.accessdata.fda.gov/drugsatfda_docs/label/>  2015/200671s000lbl.pdf |
| Empagliflozin | Anti-diabetic agent | Increased risk of lower limb amputation (mainly seen with canagliflozin in the same class), but it also has a beneficial effect in reducing cardiovascular death in patients with type 2 diabetes. | <https://www.accessdata.fda.gov/drugsatfda_docs/label/2>  023/204629s042lbl.pdf |

# References

Burridge, P.W., Matsa, E., Shukla, P., Lin, Z.C., Churko, J.M., Ebert, A.D., et al. (2014). Chemically defined generation of human cardiomyocytes. *Nat Methods* 11(8)**,** 855-860. doi: 10.1038/nmeth.2999.

Feyen, D.A.M., McKeithan, W.L., Bruyneel, A.A.N., Spiering, S., Hörmann, L., Ulmer, B., et al. (2020). Metabolic Maturation Media Improve Physiological Function of Human iPSC-Derived Cardiomyocytes. *Cell Rep* 32(3)**,** 107925. doi: 10.1016/j.celrep.2020.107925.

Huebsch, N., Charrez, B., Neiman, G., Siemons, B., Boggess, S.C., Wall, S., et al. (2022). Metabolically driven maturation of human-induced-pluripotent-stem-cell-derived cardiac microtissues on microfluidic chips. *Nature Biomedical Engineering* 6(4)**,** 372-388. doi: 10.1038/s41551-022-00884-4.

Ng, E.S., Davis, R., Stanley, E.G., and Elefanty, A.G. (2008). A protocol describing the use of a recombinant protein-based, animal product-free medium (APEL) for human embryonic stem cell differentiation as spin embryoid bodies. *Nat Protoc* 3(5)**,** 768-776. doi: 10.1038/nprot.2008.42.

Zhang, J.Z., Zhao, S.R., Tu, C., Pang, P., Zhang, M., and Wu, J.C. (2021). Protocol to measure contraction, calcium, and action potential in human-induced pluripotent stem cell-derived cardiomyocytes. *STAR Protoc* 2(4)**,** 100859. doi: 10.1016/j.xpro.2021.100859.
